# Phytochemical and Ecological Analysis of Two Varieties of Hemp (*Cannabis sativa* L.) Grown in a Mountain Environment of Italian Alps

**DOI:** 10.3389/fpls.2019.01265

**Published:** 2019-10-15

**Authors:** Radmila Pavlovic, Sara Panseri, Luca Giupponi, Valeria Leoni, Cinzia Citti, Chiara Cattaneo, Maria Cavaletto, Annamaria Giorgi

**Affiliations:** ^1^Centre of Applied Studies for the Sustainable Management and Protection of Mountain Areas–CRC Ge.S.Di.Mont., University of Milan, Edolo, Italy; ^2^Department of Agricultural and Environmental Sciences–Production, Landscape and Agroenergy–DISAA, University of Milan, Milan, Italy; ^3^Department of Veterinary Science and Public Health–VESPA, Università degli Studi di Milano, Milan, Italy; ^4^Department of Life Sciences, University of Modena and Reggio Emilia, Modena, Italy; ^5^Department of Sciences and Technological Innovation–DiSIT, Università del Piemonte Orientale, Vercelli, Italy

**Keywords:** Futura 75, Finola, plant metabolomics, Terpenes, functional strategy

## Abstract

Hemp (*Cannabis sativa* L.) is a multifunctional crop that is capable of prompt environmental adaptation. In this study, a monoecious cultivar (Futura 75) and a dioecious one (Finola) were tested in a mountain area in Valsaviore (Rhaetian Alps, Italy; elevation: 1,100 m a.s.l.) during the growing season 2018. Phytochemical behavior was evaluated by different analytical approaches: HPLC-high-resolution mass spectrometry, SDS-PAGE LC-MS/MS, HS-SPME GC-MS, and GC-FID in order to obtain complete profile of two varieties cultivated in altitude. CSR functional strategy used for ecological evaluation revealed that both genotypes are mainly competitors, although Finola is more stress tolerator (C:S:R = 57:26:17%) than Futura (C:S:R = 69:15:16%). The Finola inflorescences were characterized by higher quantities of β-ocimene and α-terpinolene, while α- and ß-pinene accompanied by extremely high ß-myrcene were found as predominant in Futura. Both varieties were rich in sesquiterpenes (45 recognized) among which *trans*-caryophyllene and α-humulene were the most abundant. Total tetrahydrocannabinol level was lower than 0.1%, while the most abundant cannabinoid was cannabidiolic acid (CBDA): 2.3% found in Finola vs. 2.7% revealed for Futura. The level of corresponding neutral form, cannabidiol, varied drastically: 0.27% (Finola) vs. 0.056% (Futura). Finola showed the unique cannabinoid profile with unexpectedly high cannabidivarin, 2-fold higher that corresponding acidic analogue, whereas the particularity of Futura 75 was the occurrence of cannabigerolic acid (CBGA) in the quantities that was double than those exposed for Finola. The seeds from both chemovars proved to be rich in polyunsaturated fatty acids, and Finola showed a higher ratio ω6/ω3. No difference was found in the protein content, and the SDS-PAGE profile was similar. The most abundant protein was edestin, followed by heat shock protein 70, ß-conglycinin, and vicilin. In conclusion, comprehensive phytochemical and ecological study of two fiber-type varieties cultivated in Italian Alps displayed specific, legal, and safe cannabinoids profile, followed by particular terpene composition, polyunsaturated fatty acids content, and favorable protein profile. This postulates that geographical provenience of hemp should be considered in selecting a variety that would be suitable for a specific end-use nutraceutical application.

## Introduction

Hemp (cannabis, *Cannabis sativa* L.) has been emerging as a resourceful plant that is highly adaptable to the most of European climate and geographical conditions (Salentijn et al., 2015). Plenty of advantageous ecological, agronomical, and pharmaceutical properties that this multifunctional crop possesses qualifies it as a convenient raw material for various traditional (fiber, food, oil, medicine) or innovative industrial application (new biomaterials and biofuels) (Amaducci et al., 2015, Bonini et al., 2018). A modest, non-demanding cultivation accompanied by a sustainability of cannabis-derived products are the main reasons of its evident agronomic expansion. Historically, hemp was frequently grown in 1930s/40s mainly for the production of technical textiles, but despite its versatility, the cultivation of hemp was prohibited in the beginning of the 1950s by reason of problematic presence of psychoactive substance Δ-9-tetrahydrocannabinol (THC) that is produced by some hemp varieties. Nowadays, this has been partly abolished and the European Union permits the cultivation of hemp with THC content being less than 0.20% (EU Regulation, 2013) In Italy, regulation published on 14th of January 2017 delineates the conditions for hemp production, its commercialization and utilization in for specific industrial purposes (Legge 242/2016). Different genotypes have been selected and registered along with standardized cultivation methods.

The uniform taxonomy of the *Cannabis sativa* L. has been proven rather challenging and often confusing, due to the huge variability within the same genus (McPartland, 2018). Recently, a simple and practical classification into few different chemotypes on the base of the cannabinoids profile has been proposed (Aizpurua-Olaizola et al., 2016). However, two main phenotypes according to THC content are most frequently taken into consideration: the first one is drug-type cannabis with high THC amount issued for medical and recreational purposes and the second one is fiber-type (industrial) hemp with THC less than 0.2%.

*Cannabis sativa* L. is naturally dioecious, with the staminate plants that are usually slender, taller, and that come to flower earlier that the pistillate ones. Hemp is wind pollinated, and the male plants die after producing millions of pollen grains. A small percentage of monoecious plant can naturally occur, particularly in short-day conditions. Monoecious varieties have been selected in modern times to reduce the agronomic problems related to the sexual vegetative dimorphism present in dioecious varieties, in particular the lack of an efficient mechanization for harvesting the seeds, and the lower fiber quality and yield losses encountered when harvesting dioecious varieties at seed maturity (Faux et al., 2013). Usually seeds in monoecious varieties are smaller than in dioecious ones.

Currently, the European Union has regulated commercial production and distribution of about 70 hemp varieties (Plant Variety Catalogues, Databases & Information Systems, 1995). Among all those varieties, two are particularly spread in Italy: Finola and Futura 75. Finola is dioecious, an auto-flowering hemp variety with a short stature, adaptation to high latitudes, high yield, and it is presently one of the most popular seed cultivar (Schluttenhofer and Yuan, 2017). Futura 75 (French monoecious) has been the most cultivated hemp variety in Italy in the last 5 years (Frassinetti et al., 2018). Generally, monoecious variates are a result of long breading efforts and are driven by the search of tall stalks that would give a high fiber output.

As mentioned previously, industrial hemp has been traditionally cultivated as a source of fibers but increasing concern in the nutritional properties of the seeds has promoted its further development, especially for the fatty acid (Callaway, 2004) and protein portions (Tang et al., 2006). Furthermore, there is a growing interest about the valorization of a hemp inflorescence that could display potential pharmacological effects (Amaducci et al., 2015). To this regard, hemp essential oil is reported to have an intriguing antimicrobial activity, whereas the whole decocted plant is used against migraine, or as pain-relieving substance (Zengin et al., 2018, Bonini et al., 2018).

Hemp is also a prolific producer of bioactive secondary metabolites, and their recovery from inflorescences contributes to identification of this plant as a multipurpose crop. The most important secondary metabolites are phytocannabinoids that have received attention owing to their biomedical relevance. Acidic forms of cannabinoids are exclusively biosynthesized in the trichromes: inflorescences of industrial hemp varieties are particularly rich in cannabidiolic acid (CBDA) that is susceptible to the spontaneous decarboxylation to cannabidiol (CBD) under favorable environmental/conservational conditions, such as heat and light. CBD is responsible for a variety of pharmacological actions that could have some remarkable applications, but unlike THC, CBD does not possess any psychoactive effects (Russo, 2011). That is the reason why the CBD dietary supplements obtained from different industrial cannabis chemotypes have become particularly widespread (Pavlovic et al., 2018).

**Figure 1:**
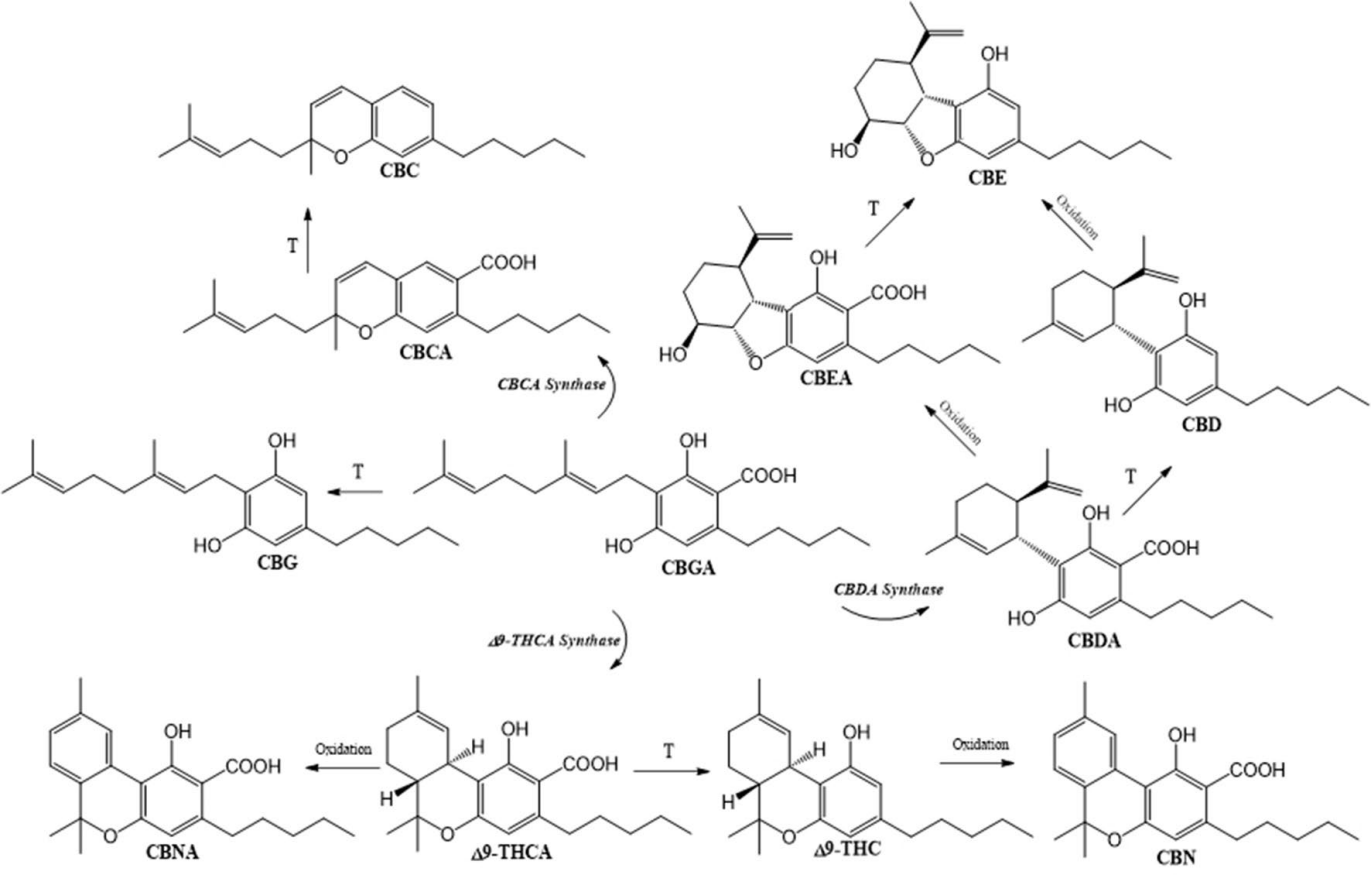
Biosynthesis of main phytocannabinoids. Cannabigerolic acid (CBGA), synthetized from geranyl diphosphate and olivetolic acid, is the central precursor of tetrahydrocannabinolic acid (Δ9-THCA), cannabidiolilc acid (CBDA), and cannabichromenic acid (CBCA), which contain an n-pentyl side chain. Decarboxylation of acidic precursors gives respectively Δ9-tetrahydrocannabinol (Δ9-THC), cannabidiol (CBD), and cannabichromene (CBC). Cannabinol (CBN) and cannabinolic acid (CBNA) are formed by non-enzymatic oxidation of THC(A), while cannabielsoin (CBE) and cannabielsoinic acid (CBEA) are produced by intramolecular CBD(A) modifications.

Although CBD and THC are the key molecules, the plant itself is capable of generating a whole series of phytocannabinoids: about 120 have been isolated to date (ElSohly et al., 2017). Based on the diversity of their structure, phytocannabinoids are classified into 11 general types (Hanuš et al., 2016). The biosynthetic pathway of the most abundant members of the phytocannabinoid class (with appurtenant enzymes involved) is presented in the **Figure 1**. This metabolic sequence includes the production of central precursor cannabigerolic acid (CBGA) that is synthesized from geranyl diphosphate and olivetolic acid. The activities of specific synthases lead to the production of tetrahydrocannabiolic acid (THCA), CBDA, and cannabichromenic acid (CBCA). Corresponding chemically neutral, but physiologically active counterparts are produced following decarboxylation in order to generate cannabigerol (CBG), THC, CBD, or cannabichromene (CBC). Other phytocannabinoids detected in plant samples include principal oxidation products of THC(A) and CBD(A): cannabinol (CBN) and cannabinolic acid (CBNA) obtained from THC(A) and cannabielsoin (CBE) and cannabielsoinic acid that derive from CBD(A) (**Figure 1**). Furthermore, the important phytocannabinoids family, so-called “cannabivarin” class, that regularly accompanies the main ones, is produced from condensation of geranyl diphosphate with divarinic acid, which results in a propyl instead of the pentyl side chain.

Although the attention of scientific community has been focused on major phytocannabinoids, the phytochemical characterization of cannabis highlights the presence of various non-cannabinoids constituents including flavonoids, spiroindans, dihyrostilbenes, dihydrophenanthrenes, lignanamides, steroids, and alkaloids (Pollastro et al., 2018). Their characterization is scarce and random, especially when the inflorescences of industrial hemp is concerned.

On the other hand, one non-phytocannabinoid class that is studied in much more details is terpene category. They represent the volatile component that has been claimed to have a synergic action with cannabinoids (Russo, 2019). Hemp plants produce and accumulate a terpene-rich resin in glandular trichomes, which are abundant on the surface of the female inflorescence. Bouquets of different monoterpenes and sesquiterpenes are important components of cannabis resin as they define some of the unique organoleptic properties and may also influence medicinal qualities of different cannabis strains and varieties (Lewis et al., 2018).

Choosing a genotype suitable for a specific end-use application and adapted to an environment is of paramount importance to the success of hemp cultivation. Hemp is a plant adaptable to various growing and ecological conditions, but there is no data available in literature that concern the mountain environment. A higher altitude could affect the secondary metabolites profile of flowers (cannabinoids and terpenes) and main nutritional components of seeds (fatty acids and proteins). In this research, a monoecious cultivar, Futura 75, and a dioecious one, Finola, were studied in a mountain environment of Italian Alps (Valsaviore, 1100 m a.s.l.) during the growing season 2018 for their potential to provide nutraceutical substances and to study their behavior from an ecological and phytochemical point of view to assess their usefulness as mountain crop. The special attention was given to the inflorescences that were studied by new metabolomic, untargeted analytical approach by means of high-resolution mass spectrometry (HRMS), which enabled the detection of a whole series of secondary metabolites.

## Materials and Methods

### Experimental Fields and Sample Collection

Valsaviore is an alpine valley on the orographic left of the upper-middle Valle Camonica; experiments were carried out on two terraced fields of two local farms (Dimensione Natura and Shanty Maè) in the municipality of Saviore dell’Adamello (latitude 46°4’53”04 N, Longitude 10°24’2”52 E, elevation 1,100 m a.s.l.) during the season 2018. This area belongs to the Temperate Oceanic bioclimate (Rivas-Martinez and Rivas-Saenz, 2009). It has a rainfall of 1.100 mm per year, concentrated mainly in the spring and the autumn; the annual average temperature is about 8,9°C; minimum temperatures and precipitation are during the winter months (data source: Centro Meteo Lombardo). According to Blasi et al. (2014), the area is within the Northeastern Alps Ecoregional Subsection (Central and Eastern Alps Section, Alpine Province, Temperate Division).

The experimental fields were obtained from terraced mountainside, from abandoned fields that had not been cultivated for more than 60 years. Thus, fertilization was not done, and soil was prepared ploughing with an excavator at depth 70–80 cm without soil tipping, manual removal of weed roots, and successive mechanical milling. No irrigation supplies were needed after sowing either in summer period thanks to the natural soil water availability. Plants were maintained under identical fertilization conditions throughout the field experiments. Finola (FINOLA DE 166 -2700754 11-2016) and Futura 75 (FUTURA 75 FR 484520 AA COD. B 174613 02/2018) seeds were donated by Hemp Farm Italia in February 2018.

The two varieties were planted in six randomized blocks: 3 blocks of Finola variety plus 3 blocks of Futura 75 (for final surface of about 130 m^2^ for each variety) in the two terraced fields; in both cases, Finola had a planting pattern of 10 cm between rows and at intervals of 10 cm within the row, while Futura 75, a higher and larger variety, requested a more spacious planting pattern of 20 cm between rows and at intervals of 20 cm within the row. Crop was protected against weeds by frequent hand weeding, but no pesticides were supplied.

Finola seeds were sown the 23^rd^ of May 2018, while Futura 75 seeds were sown the 30^th^ of May 2018 in both farms. The seeds were sown with manually 3–4 inches deep. It was decided to use experimental fields from Dimensione Natura farm to concentrate data analysis about inflorescences, while experimental fields from Shanty Maè farm were designated to obtain seeds.

For both cultivars, it took about 5 days to obtain over the 50% germination of seeds. In the Dimensione Natura farm, male plants of dioecious cultivar Finola were eliminated (to obtain the maximum concentration of secondary metabolites in the inflorescences trichomes) starting from the third week from sowing (15^th^ of June 2018) and removing them every 3 days. Male plants were estimated being about 50% of total plants and reached a final height from 15 to 70 cm (measured on 30 plants randomized) before being removed. The final crop density, considering removing males and other factors as snails and mice, was about 10 plants per m^2^. The height of Finola female plants was calculated on a randomized sample of 30 plants (**Table 1**).

**Table 1:** Height of Finola and Futura 75 plants for inflorescence harvesting (average of 30 plants randomly selected).

Day		15	30	45	60	75	90
**Height of plants (cm)**	***Finola***	15	40	100	120	150	/
	***Futura 75***	30	70	100	150	230	300

Futura 75, as monoecious plant, started producing male flowers the second week of August, after the complete flowering of Finola. No plants were removed, and the final crop density was about 20 plants per m^2^ in both experimental fields. Futura 75 male and female flowers appeared from the second week of August until the second week of September. The male flowers of Futura were also removed in Dimensione Natura farm, but it was not possible to completely avoid some pollination. Visual estimation of flowering calendar for both chemotypes is presented in **Table 2**.

**Table 2:** Flowering calendar for Finola and Futura.

Percentage of flowering		10%	50%	100 (full flowering)	Inflorescence harvesting
Issue date female inflorescences	***Finola***	01/07/2018	07/07/2018	12/07/2018	06/08/2018
	***Futura 75***	15/08/2018	30/08/2018	15/09/2018	28/09/2018

Harvest of inflorescences was carried out at flowering, corresponding to the phenological codes 2202 and 2302 (Mediavilla et al., 1998) for dioecious and monoecius varieties, respectively. The fresh inflorescences were manually sampled from the same plants, cutting the 30 cm upper part of the stem, from 10 to 20 plants per plot randomly chosen. Then they were left to air-dry, protected from light in open containers on room temperature (25°C) for 2 weeks (Hillig, 2004). Afterwards, dried inflorescences were collected, placed in the plastic bags, put under the vacuum, and stored in cool room until analysis. Low temperature was kept, avoiding as much as possible changes in metabolites, as cannabinoids and terpenes.

Harvest of seeds was performed at the start of September for Finola and start of October for Futura 75 in Shanty Maè farm. The seeds were manually sampled cutting the 30 cm upper part of the stem of every plant (phenological codes: 2204 and 2306, Mediavilla et al., 1998), then left to dry in a cool and dry room for 2 weeks. Seeds were then sieved, and a random sample of about 30 grams of each variety was chosen for laboratory tests.

### Functional Strategy

The analysis of the CSR (C—competitors, S—stress-tolerators, R—ruderals) functional strategy of Grime, 1974, Grime, 1977, Grime, 2001 of the two varieties of *Cannabis sativa* L. was performed according to the method proposed by Pierce et al. (2017). In detail, 10 fully expanded leaves for each variety were collected in July 2018. The leaf samples were wrapped in moist tissue paper and stored in the dark overnight at 4°C. Leaf fresh weight (LFW) was determined from these saturated organs using analytical balance Precisa XB 220A and the leaf area (LA) was measured using ImageJ software (Schneider et al., 2012) after scanning the leaves with high resolution digital scanner (hp Scanjet 3670). Leaf dry weight (LDW) was measured after oven drying at 105°C for 24 h. CSR values and functional strategy were determined using “StrateFy” tool (Pierce et al., 2017). Finally, CSR coordinates were projected in the CSR ternary graph using the “ggplot2” package of R (R Development Core Team, 2018) and one-way ANOVA was performed considering C, S, and R values as dependent variables and varieties as independent variables.

### Seed Weight and Proteins Analysis

The seed weight was assessed by weighing (using analytical balance Precisa XB 220A) a sample of 50 seeds per genotype. The test was performed for the commercial seeds and for the seeds obtained from the experimental fields of both varieties and was done in triplicate.

Hempseed (1 g) were ground in a mortar at 4°C, added with 20 ml of a solution of 10% trichloroacetic acid (TCA) in cold acetone (−20°C), containing 20 mM dithiothreitol and 1% protease inhibitors cocktail (Sigma), filtered, and incubated overnight at −20°C. Seed protein precipitate was obtained by centrifugation (18,000g, 1 h, 4°C), and the pellet was washed (three times) with acetone and dried. The protein precipitate was extracted with a solution of 7 M urea, 2 M thiourea, 4% w/v CHAPS, 100 mM DTT, IPG-buffer (pH 3–10). Protein content was estimated by Bradford (1976).

Protein characterization was realized performing sodium dodecyl sulphate–polyacrylamide gel electrophoresis (SDS-PAGE) and direct protein identification by LC-MS/MS analysis. For SDS-PAGE, 10 µg of hempseed proteins were mixed with Laemmli buffer (2% w/v SDS, 10% glycerol, 5% b-mercaptoethanol,62 mM Tris-HCl pH 6.8), boiled for 5 min, and loaded on 10 × 8 cm vertical 12% polyacrylamide gels. SDS-PAGE was performed at 15 mA for 30 min and 30 mA for 3 h at 10°C with a Mini Protean II Xi System (Bio-Rad). The running buffer was 25 mM Tris-HCl, 200 mM glycine, 0.1% w/v SDS. Gels were stained with Colloidal Coomassie brilliant blue G250 (Bio-Rad Laboratories).

Protein bands of interest were manually excised and in-gel trypsin digested as described in Spertino et al. (2012). Proteins were identified by liquid chromatography tandem mass spectrometry (LC-MS/MS) analysis by a micro-LC Eksigent Technologies (Dublin, USA) system (Spertino et al., 2018). Briefly, the mass spectrometer worked in information dependent acquisition (IDA) mode. MS data were acquired with Analyst TF 1.7 (ABSCIEX, Concord, Canada). The mass spectrometry files were searched using Mascot v. 2.4 (Matrix Science Inc., Boston, MA, USA). A search tolerance of 0.6 Da was specified for the peptide mass tolerance, and 100 ppm for the MS/MS tolerance.

### Seeds Fatty Acid Composition

Seed samples of the investigated varieties were grinded using superfine grinding extractor—intensive vibrational mill (Model MM400, Retsch GmbH, Haan, Germany). To obtain a representative seed powder, a 50 ml jar with 20 mm stainless steel balls at a frequency of 25 Hz for 1 min was used.

Lipid extraction (Bligh and Dyer, 1959) was performed using 7.0 g of powdered seeds. The seed oil was extracted by a Soxhlet extractor and petroleum ether for 6 h at 60°C. n-Hexane was used as the solvent, and following the extraction method oil was separated from n-hexane using a rotator apparatus. The fatty acid composition of hemp seeds was determined using GC. In this method, the fatty acids were turned volatile using the method of methyl esterification (Metcalf et al., 1996). The prepared solution was injected into a GC Trace Ultra (ThermoFisher Scientific) equipped with a flame ionization detector (FID) detector, with the following specifications. Capillary column RTX-2560 (100 m × 0.25 mm id, 0.20 μm); the carrier gas was nitrogen, with the purity of 99.9%. The injector and the detector temperature were equal to 260 and 280°C, respectively. The oven temperature was kept at 100°C for 5 min and increased to 240°C at the rate of 4°C per minute and maintained at 240°C for 30 min (Tang et al., 2015; Zhang et al., 2015). The chromatographic profiles of analyte were elaborated with an Azur Software (Analytical Technology, Brugherio, Italia). Identification and quantitative evaluation of fatty acids was realized confronting retention times and areas with the ones of standard mixes FAMEs (fatty acid methyl esters). All analyses were done in three biological replicates.

### Inflorescence Analysis

#### Chemical and Reagents

All HPLC or analytical grade chemicals were from Sigma (Sigma–Aldrich, St. Louis, MO, USA). Formic acid 98–100% was from Fluka (Sigma–Aldrich, St. Louis, MO, USA). Ultrapure water was obtained through a Milli-Q system (Millipore, Merck KGaA, Darmstadt, Germany). For head-space (HS) analysis, the SPME coating fiber (DVB/CAR/PDMS, 50/30 µm) was from Supelco (Bellefonte, PA, USA). Acetonitrile, 2-propanol, and formic acid LC-MS grade were purchased from Carlo Erba (Milan, Italy). CBD, THC, CBN, CBG, CBC, cannabidivarin (CBDV), tetrahydrocannabivarin (THCV), CBDA, THCA, CBNA, CBGA, CBCA, cannabidivarinic acid (CBDVA), and tetrahydrocannabivarinic acid (THCVA) were purchased from Sigma Aldrich (Round Rock, Texas). All cannabinoids were analytical standards at concentration 1 mg ml^−1^ delivered as solutions in methanol.

#### Superfine Grinding (SFG) Sample Preparation

Superfine Finola and Futura 75 inflorescence powder was prepared using mechanical grinding-activation in an energy intensive vibrational mill. Five biological replicates (1.0 g each) were ground in a high intensity planetary mill. The mill was vibrating at a frequency of 25 Hz for 1 min, using two 50 ml jars with 20 mm stainless steel balls. Prior to use, jars were precooled with liquid nitrogen. The speed differences between balls and jar result in the interaction of frictional and impact forces, releasing high dynamic energies. The interplay of all these forces results in the very effective energy input of planetary ball mills. Mechano-chemical technology has been developed and successfully adopted in different fields (synthesis of superfine powder, surface modification, drug and pharmaceutical applications) and could represent a novel research tool.

#### Accelerated Solvent Extraction (ASE) for Cannabinoid Profiling

The extraction procedure was done according to the already published procedure (Calvi et al., 2018a, Calvi et al., 2018b). Briefly, all extractions to delineate the cannabinoid profile were performed by accelerated solvent extraction apparatus using an ASE 350 (Thermo-Fisher Scientific, Waltham, MA, USA) with 34-ml stain steel cells. Inflorescence powder (100 mg) obtained by using SFG was weighed and then homogenized with an equal weight of diatomaceous earth and transferred into the cell. Then, 100 μl of extraction solution containing the IS (diazepam 1 mg ml^−1^) was added. Afterwards, the remaining empty part of the cell was filled-up with diatomaceous earth. Room temperature of 25°C, pressure (1500 psi), number of static cycles (2 cycles, 5 min each), purging time (60 s with nitrogen), and rinse volume (90%) were used for the study. Organic extracts (25 ml) were obtained using pure methanol and were dried under vacuum in a centrifugal evaporator. The residue was dissolved in 1 ml of acetonitrile, and after proper dilution (1:10) in starting mobile phase, 2 μl were submitted to analysis by HPLC-Q-Exactive-Orbitrap-MS. To obtain the matrix-matched calibration curves, blank samples (100 mg of commercially available officinal plants mixture previously analyzed for the absences of cannabinoids) were used and spiked with appropriate standard solution of 14 commercially available cannabinoids listed above covering the two concentration range from 0.1 to 10 μg g^−1^ and from 10 to 1000 μg g^−1^.

#### Cannabinoids LC-Q-Exactive-Orbitrap-MS Analysis

The cannabinoid profile in both cultivars was evaluated applying the method recently published by us (Pavlovic et al., 2018) with modification that was essential for the untargeted analysis. In order to perform HPLC-Q-Exactive-Orbitrap®-MS analysis, samples extracted with ASE were prepared as specified in paragraph 2.5.3. Chromatography was accomplished on an HPLC Surveyor MS quaternary pump, a Surveyor AS autosampler with a column oven, and a Rheodyne valve with a 20 μl loop system (Thermo Fisher Scientific, San Jose, CA, USA). Analytical separation was carried out using a reverse-phase HPLC column 150 × 2 mm i.d., 4 μm, Synergi Hydro RP, with a 4 × 3 mm i.d. C18 guard column (Phenomenex, Torrance, CA, USA). The mobile phase was run as a gradient that consisted of water and acetonitrile both acidified with 0.1% formic acid. The gradient was initiated with 95% eluent 0.1% aqueous formic acid with a linear decrease up to 95% in 30 min. The mobile phase was returned to initial conditions at 35 min, followed by a 5-min re-equilibration period. This condition was maintained for 5 min. The flow rate was 0.3 ml/min. The column and sample temperatures were 30°C and 5°C, respectively. The mass spectrometer Thermo Q-Exactive Plus (Thermo Scientific, San Jose, CA, USA) was equipped with a heated electrospray ionization (HESI) source. Capillary temperature and vaporizer temperature were set at 330 and 380°C, respectively, while the electrospray voltage operating in positive was adjusted at 3.30 kV. Sheath and auxiliary gas were 35 and 15 arbitrary units, with S lens RF level of 60. The mass spectrometer was controlled by Xcalibur 3.0 software (Thermo Fisher Scientific, San Jose, CA, USA). The exact mass of the compounds was calculated using Qualbrowser in Xcalibur 3.0 software. The FS-dd-MS^2^ (full scan data-dependent acquisition) in positive mode was used for both screening and quantification purposes. Resolving power of FS adjusted on 70,000 FWHM at m/z 200, with scan range of *m/z* 100–900. Automatic gain control (AGC) was set at 3e^6^, with an injection time of 200 ms. A targeted MS/MS (dd-MS^2^) analysis operated in both positive and negative mode at 35,000 FWHM (*m/z* 200). The AGC target was set to 2e^5^, with the maximum injection time of 100 ms. Fragmentation of precursors was optimized as three-stepped normalized collision energy (NCE) (20, 40, and 40 eV). Detection was based on retention time and on calculated exact mass of the protonated molecular ions, with at least one corresponding fragment of target compounds (Pavlovic et al., 2018). Good peak shape of extracted ion chromatograms (EICs) for targeted compounds was ensured by manual inspection, as well.

#### Untargeted Metabolomics Approach

Raw data from Xcalibur 3.0 software were processed with Compound Discoverer™ (Thermo Scientific). In particular, this platform applies peak detection, retention time, profile assignment, and isotope annotation. A list of potential compounds was suggested for each chromatographic peak depending on the mass fragmentation of the parent pseudomolecular ion. Accurate mass determination generating elemental composition within a narrow mass tolerance window for identification based on accurate precursor mass. For some signals, the putative identification was confirmed by analysis performed on authentic standard. Metabolite identification was based on accurate mass and mass fragmentation pattern spectra against MS-MS spectra of compounds available on mzCloud database (HighChem LLC, Slovakia). The ChemSpider Web services platform was used as additional confirmation tool. If mass fragmentation pattern did not correspond to any of databases annotated by Compound Discoverer™ software, manual confirmation of their fragments was performed.

#### HS-SPME and GC-MS Analysis for Terpenes Examination

Exhaustive analytical procedures were described in detail in our recently published article (Calvi et al., 2018b). In brief, inflorescence powder (100 mg) previously grinded was weighed and put into 20 ml glass vials along with 100 μl of the IS (4-metil-2-pentanone, 20 mg/ml in 2-propanol). Each vial was fitted with a cap equipped with a silicon/PTFE septum (Supelco, Bellefonte, PA, USA). To keep the temperature constant during analysis (37°C), the vials were maintained in a cooling block (CTC Analytics, Zwingen, Switzerland). At the end of the sample equilibration time (30 min), a conditioned (60 min at 280°C) SPME fiber was exposed to the headspace of the sample for 120 min using a CombiPAL system injector autosampler (CTC Analytics, Zwingen, Switzerland).

Analyses were performed with a Trace GC Ultra coupled to a Trace DSQII quadrupole mass spectrometer (MS) (Thermo-Fisher Scientific, Waltham, MA, USA) equipped with an Rtx-Wax column (30 m × 0.25 mm i.d. × 0.25 µm film thickness) (Restek, Bellefonte, PA, USA). The oven temperature program was: from 35°C, held for 8 min, to 60°C at 4°C/min, then from 60 to 160°C at 6°C/min and finally from 160 to 200 at 20°C/min. Helium was the carrier gas, at a ﬂow rate of 1 ml/min. Carry over and peaks originating from the fibers were regularly assessed by running blank samples. After each analysis, fibers were immediately thermally desorbed in the GC injector for 5 min at 250°C to prevent contamination. The MS was operated in electron impact (EI) ionization mode at 70 eV. An alkanes mixture (C8-C22, Sigma R 8769, Saint Louis, MO, USA) was run under the same chromatographic conditions as the samples to calculate the Kovats Retention Indices (RI) of the detected compounds (Giorgi et al., 2012, Giorgi et al., 2013a; Giorgi et al., 2015). The mass spectra were obtained by using a mass selective detector, a multiplier voltage of 1456 V, and by collecting the data at a rate of 1 scan/s over the *m/z* range of 35–350. Compounds were identified by comparing the retention times of the chromatographic peaks with those of authentic compounds analyzed under the same conditions when available, by comparing the Kovats retention indices with the literature data and through the National Institute of Standards and Technology (NIST) MS spectral database. The quantitative evaluation was achieved using the internal standard procedure, and the results were finally expressed as µg/g. For both chemotype, all analyses were done in five biological replicates.

#### Statistical Analysis

Differences between two varieties for the quantitative analysis were determined using a two-tailed Student’s t-test from the BioVinci statistical program (Version 1.1.4., BioTurning Inc 2018). A p-value of less than 0.05 was considered statistically significant.

## Results

### CSR Strategy

**Figure 2:**
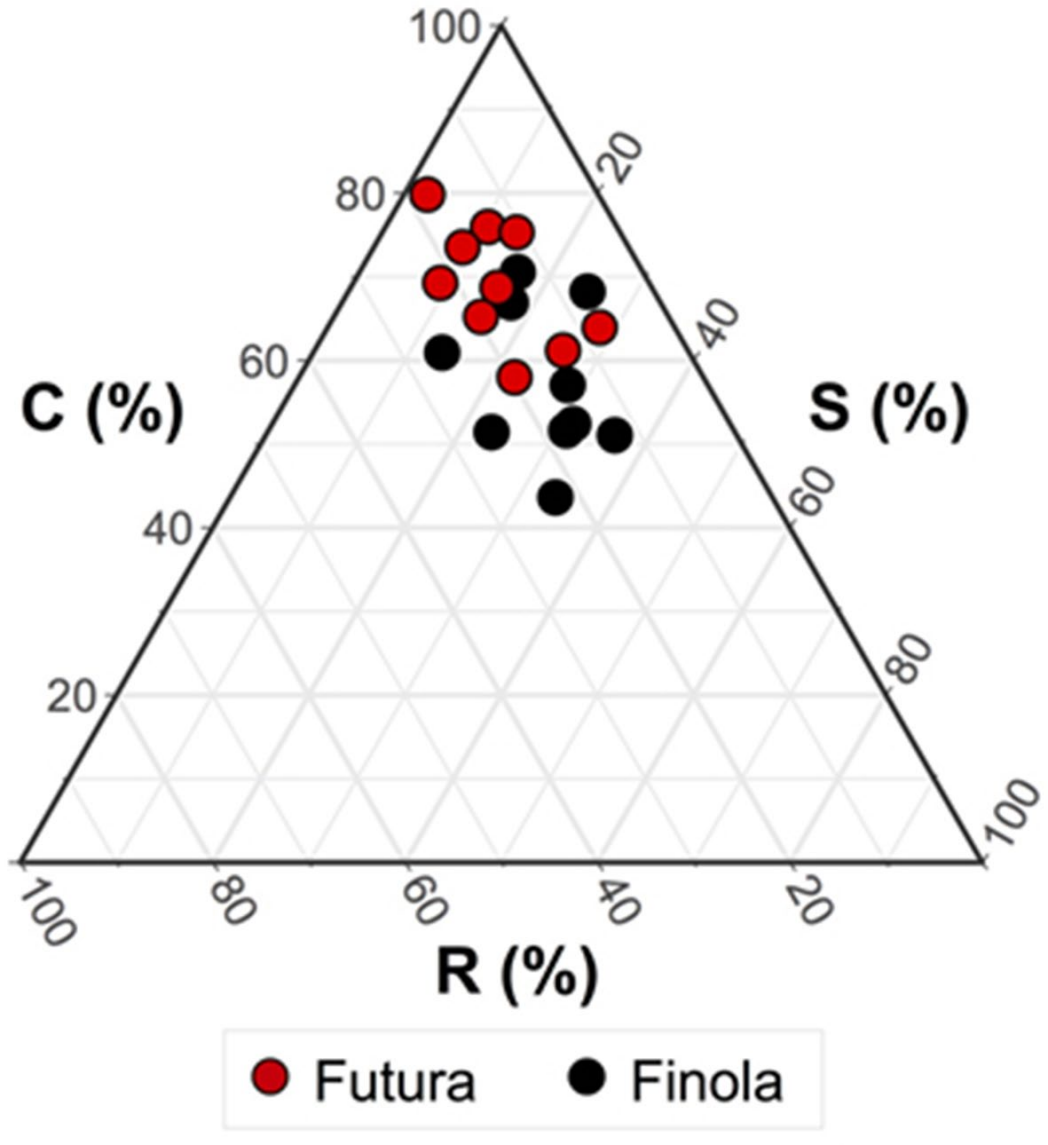
CSR classification of the two variety of *Cannabis sativa*. Mean CSR strategy of Futura 75 = C/CR (C:S:R: = 69:15:16%); mean CSR strategy of Finola = C/CSR (C:S:R: = 57:26:17%). ANOVA test revealed significant differences (*p* < 0.01) for what concerns C (competitiveness) and S (stress tolerance).

**Figure 2** reports the triangular diagram obtained by CSR analysis. Both the varieties are competitors, although Finola is more stress tolerator (C:S:R = 57:26:17%) than Futura (C:S:R = 69:15:16%), meaning for stress all those conditions able to reduce the photosynthetic activity in plants (Grime, 2001). This difference was confirmed from the results of the ANOVA test (**Table 3**) showing how the two varieties are significantly different (*p* < 0.01) for what concerns C (competitiveness) and S (stress tolerance.

**Table 3:** ANOVA results of variety effect on C, S, and R values.

Source of variance	Degrees of freedom	Sum of squares	Mean square	*F*-value	*p*-value	Sign.
C	1	684.20	684.20	10.57	0.0044	*
S	1	539.50	539.50	8.45	0.0095	*
R	1	8.60	8.59	0.31	0.582	ns

*, significant (p-value < 0.01); ns, not significant.

### Protein Yield and Characterization

**Figure 3:**
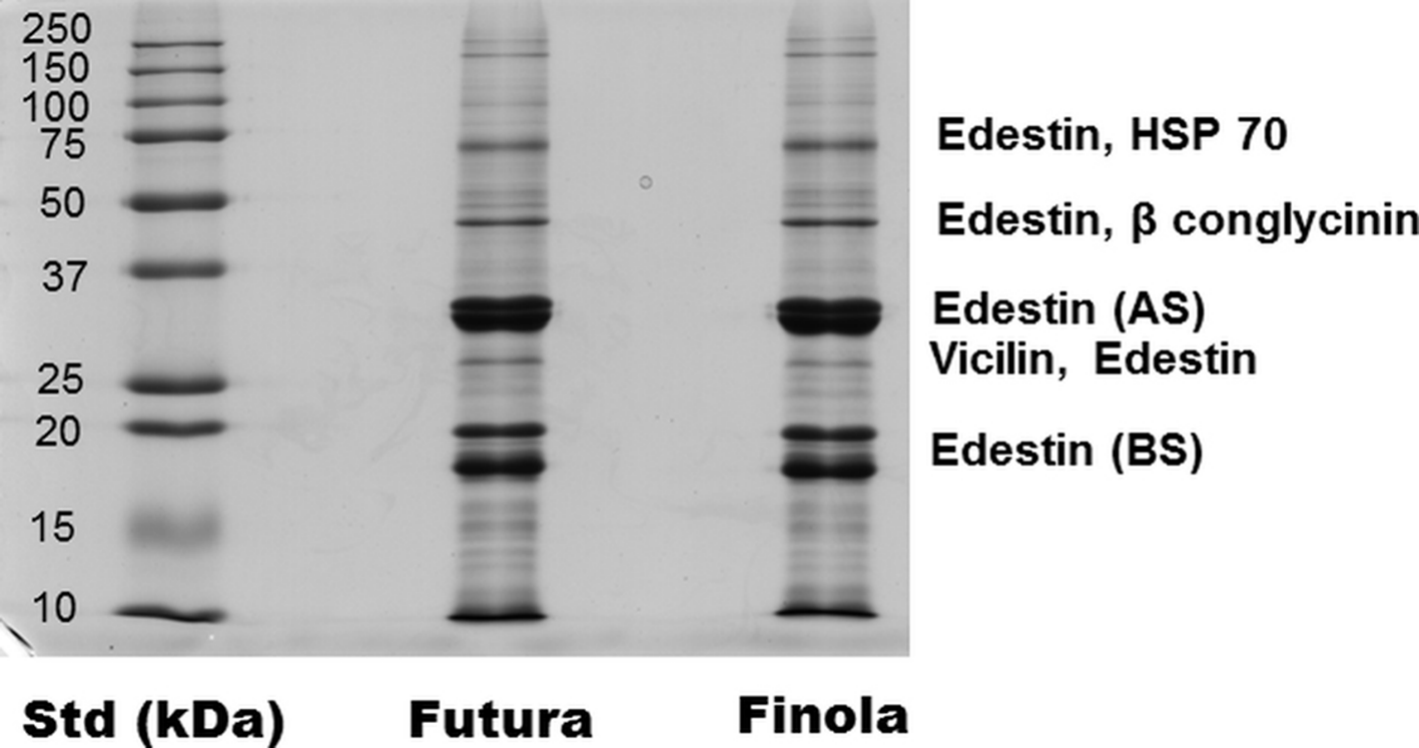
SDS-PAGE profile of hemp seed proteins. Resolved proteins were detected by Colloidal Coomassie staining and identified by MS/MS analysis.

As shown in **Table 4**, the seeds of Finola and Futura 75 are consistently smaller than the commercial seeds used to set up experimental fields. The protein yield resulted of 19.96 ± 2.20 mg/ml for Futura 75, for a total content of 39.9 mg of protein starting from 1 g of seeds. For Finola, the protein yield was of 19.69 ± 3.02 mg/ml for a total content of 39.4 mg of protein starting from 1 g of seeds. No difference was found in the protein content, and the SDS-PAGE profile was similar. The most abundant protein was the storage protein edestin, directly identified by mass spectrometry; some other proteins such as heat shock protein 70, beta conglycinin, and vicilin were also found. As shown in **Figure 3**, in fact, MS/MS analysis of the principal bands revealed the two subunits (35 and 18 kDa) of the reserved protein edestin as recently reported (Mamone et al., 2019); moreover, we can detect edestin at higher molecular weight, together with the heat shock protein (70 kDa) and conglycinin (around 50 kDa).

**Table 4:** Mean seeds weight for Futura and Finola genotypes.

50 seeds weight (g)	Mean value
Sown FUTURA	**0.923153 ± 0.008247**
Sown FINOLA	**0.617274 ± 0.016463**
Harvested FUTURA	**0.531317 ± 0.093085**
Harvested FINOLA	**0.517487 ± 0.019501**

### Seeds Fatty Acid Profile

In **Table 5**, the results of fatty acid profiling of Finola and Futura 75 seeds and the total of saturated fatty acids (SFA), monounsaturated (MUFA), polyunsaturated (PUFA), and omega-3 (ω3) and omega-6 (ω6) fatty acids are shown.

**Table 5:** Fatty acid profile and composition (w/w, %) in Finola and Futura 75 seeds.

		Finola		Futura		Statistical evaluation^a^
Fatty acids	Abbreviation	Mean	SD	Mean	SD
**Miristic**	**C14:0**	0.04	0.001	0.10	0.006	<0.001
**Miristoleic**	**C14:1**	0.01	0.001	0.06	0.000	<0.001
**Pentadecanoic**	**C15:0**	6.08	0.040	0.02	0.001	<0.001
**Palmitic**	**C16:0**	0.10	0.006	6.40	0.090	<0.001
**Palmitoleic**	**C16:1**	nd^c^	nd	0.15	0.006	<0.001
**Eptadecanoic**	**C17:0**	0.06	0.006	0.05	0.006	ns^b^
**Cis-10 eptadecanoic**	**C17:1**	0.01	0.001	nd	nd	<0.001
**Stearic**	**C18:0**	2.18	0.220	2.91	0.006	ns
**Elaidic**	**C18:1ω9t**	nd	nd	0.01	0.001	<0.001
**Oleic**	**C18:1ω9c**	9.41	0.020	11.50	0.015	<0.001
**Linolelaidic**	**C18:2ω6t**	nd	nd	nd	nd	ns
**Linoleic**	**C18:2ω6c**	57.69	0.135	57.22	0.090	ns
**Arachic**	**C20:0**	0.86	0.006	0.79	0.015	0.00176
**γ-Linolenic**	**C18:3ω6**	4.22	0.025	1.86	0.006	<0.001
**Cis-11-eicosenoic**	**C20:1**	9.26	0.875	0.37	0.006	<0.001
**Linolenic**	**C18:3ω3**	9.96	0.915	18.42	0.035	<0.001
**Eneicosanoic**	**C21:0**	0.02	0.001	0.02	0.0001	ns
**Cis-11,14-eicosenoic**	**C20:2**	0.07	0.006	0.04	0.010	0.0161
**Cis-8,11,14-eicosatrienoic**	**C20:3ω6**	0.02	0.001	0.01	0.001	<0.001
**Erucic**	**C22:1ω9**	nd	nd	0.02	0.002	<0.001
**Cis-11,14,17-eicosatrienoic**	**C20:3ω3**	nd	nd	0.01	0.001	<0.001
**Arachidonic**	**C20:4ω6**	nd	nd	0.04	0.001	<0.001
**Cis-13,16- docosadienoic**	**C22:2**	0.01	0.001	0.01	0.000	ns
**Cis-5,8,11,14,17-eicosapentanoic**	**C20:5ω3**	0.02	0.005	nd	nd	<0.001
**Nervonic**	**C24:1**	0.02	0.006	0.03	0.000	0.0104
**Saturated fatty acids (SFA)**		9.33	0.195	10.27	0.080	0.005
**Monounsaturated fatty acids (MUFA)**		18.70	0.890	12.13	0.015	<0.001
**Poliunsaturated fatty acids (PUFA)**		71.98	1.085	77.61	0.065	0.0021
**Omega-3 (ω3)**		9.97	0.920	18.43	0.035	<0.001
**Omega-6 (ω6)**		62.01	0.165	59.18	0.100	0.0028
**ω6/ω3**		6.25	0.56	3.21	0.01	<0.001

Fatty acid contents are expressed as means *±* SD (n = 3, independent biological replicates); ^a^The statistical significance, tested by t-test (two-tailed distribution) p-value less than 0.05, was considered statistically significant; ^b^nd, not detected; ^c^ns, not significant.

The principal SFA was palmitic acid (PA; 16:0) for Futura 75 (6.4%) and pentadecanoic acid (C15:0) for Finola (6.08%), then followed by stearic acid (SA; 18:0) that was in similar percentage in both varieties (the 2.18% in Finola and 2.91% in Futura 75). Also, if with a different composition, the total SFA content showed to be in analogous quantity in the two varieties (9.33% in Finola and 10.27% in Futura 75). The most abundant unsaturated fatty acids in the seeds of the two varieties proved to be linoleic acid (LA; C18:2 ω6c), in average percentage of 57.69% for Finola and 57.22% for Futura 75 and oleic acid (OA; C18:1 ω9c), and 9.41% and 11.50% for Finola and Futura 75, respectively. Another fatty acid contained in high quantity was the ω3 linolenic acid (C18:3ω3) that was found in higher quantity in Futura 75 (18.42%) compared to Finola (9.96%). Cis-11-eicosenoic acid (C20:1) was found in a significative percentage only in Finola, while only 0.37% in Futura 75. This affected the total MUFA percentage, which resulted higher in Finola (18.7%), while in Futura 75 it was 12.13%.

It is clearly shown how the seeds of both varieties are rich in PUFA, which are 71.98% for Finola and 77.61% for Futura 75. Both the genotypes are exceptionally rich source of the two essential fatty acids (EFAs) LA (18:2 ω6) and α-linolenic acid (18:3 ω3). However, as expected, the Finola showed the highest content of γ-linolenic (GLA, 4.22%), accompanied with higher average values of ω6/ω3 ratio (6.22 Finola vs. 3.21 for Futura).

### Inflorescences Analysis

#### Quantification of Cannabinoids: Targeted Metabolomics With Commercially Available Phytocannabionids

**Figure 4:**
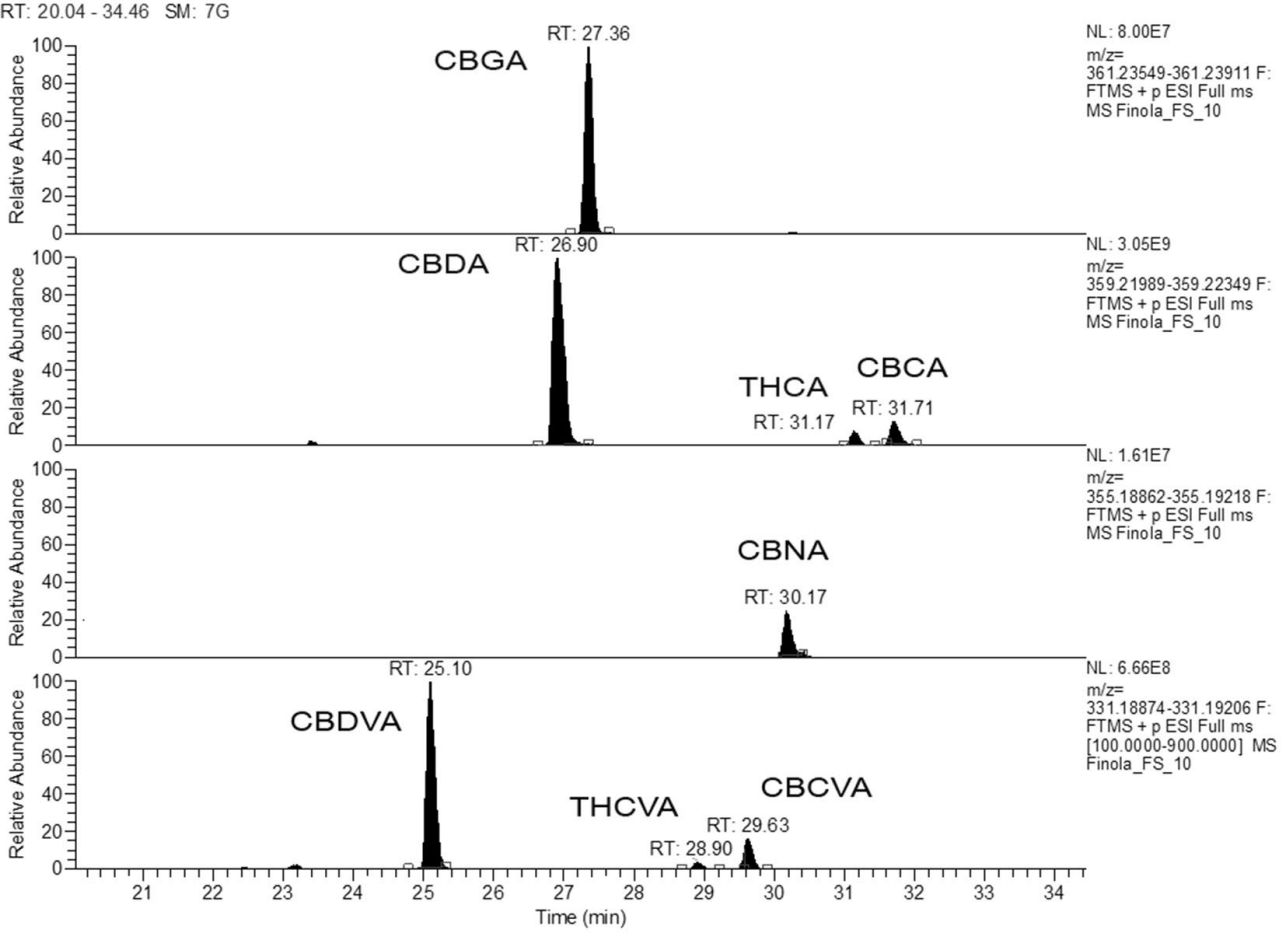
Extracted ion chromatogram for the acidic forms of phytocannabionids (CBGA, CBDA, THCA, CBDVA, and THCVA) identified according to analytical standards. The retrospective data analysis reveals the presence of CBCVA.

In the presented work, the absolute quantification of 14 cannabinoids (7 acids with appurtenant neutral counterpart) in Finola and Futura 75 inflorescences was performed. Extracted ion chromatograms (EICs) were obtained with an accuracy of 2 ppm *m/z* from total ion chromatogram engaging the *m/z* corresponding to the molecular ions. EICs of the acidic forms found in Finola chemovar are shown in **Figure 4**. According to this chromatogram, the observed relative order of elution of the detected acidic forms of pentyl phytocannabinoids is as follows: CBDA > CBGA > CBNA > Δ^9^-THCA > CBCA. This order of elution is due to increased polarity according to the increased number of polar phenolic groups (CBGA vs. Δ^9^-THCA, for example) as well as due to and formation of chromene moiety, which increased the lipophilcity of CBCA. CBDVA and Δ^9^-THCVA, the propyl homologues of CBDA and Δ^9^-THCA, respectively, have faster retention times compared to their respective pentyl analogues because of shorter (C-3) aliphatic chain. CBCA and Δ^9^-THCA, the acid precursors of CBC (RT-29.82min) and Δ^9^-THC (RT-29.43min), respectively, have longer retention times. On the contrary, CBDA and CBDVA elute before the corresponding neutral forms (CBD and CBDV), which appeared at RT-27.38min and 25.29min, respectively. This chromatographic behavior of the available standards, accompanied with characteristic mass fragmentation, enabled putative identification of additional 43 cannabinoids that express the analogous chromatographical elution behavior and analogous mass fragmentation pattern. For example, the peak that appeared at RT 29.63 was identified as CBCVA (**Figure 4**).

Based on the disposability of commercially available standards, the absolute quantification of phytocannabinoids in inflorescence extracts was performed by applying our validated method with external calibrations as it was explained in detail in experimental section (Calvi et al., 2018a; Calvi et al., 2018b). Quantitative data related to the content of phytocannabinoids in two hemp inflorescences determined by means of the HPLC-HRMS method are shown in **Table 6**. Since both varieties analyzed belong to the fiber-type hemp, it is not surprising that the most abundant phytocannabinoids were CBDA and CBD. The CBDA content did not vary drastically, although Futura inflorescence contained slightly higher concentration. On the contrary, the level of CBD was 5-times higher in Finola samples. Besides, the Finola showed the unique phytocannabinoid profile with unexpectedly high CBDV, twice higher than the corresponding acidic form (CBDVA). Futura exhibited the same trend regarding the “varin” phytocannabinoids, although not as accentuated as for Finola. Both varieties contained the neglected levels of THCA and THC, with corresponding C3 analogues detected under the limit of quantification (LOQ). The content of CBNA especially in Futura should not be underestimated, as CBNA is formed from THCA by non-enzymatic oxidation. Characteristically, Finola presented the small amount of CBCA, whereas the particularity of Futura was the occurrence of CBGA in the quantities that was double that those exposed for Finola.

**Table 6:** Phytocannabinoids content (µg/g) in investigated hemp inflorescences (average ± SD, n = 5 independent biological replicates).

	FINOLA		FUTURA		Statistical significance
	Mean	SD (±)	Mean	SD (±)	
**Neutral forms**					
**CBD**	2,614	58	561	49	<0.001
**Δ^9^-THC**	299	14	212	22	0.005
**CBN**	12	2	58	12	0.005
**CBC**	<LOQ^a^	/	69	5	<0.001
**CBG**	19	8	45	9	<0.001
**CBDV**	4,888	126	1,804	129	<0.001
**Δ^9^-THCV**	<LOQ	/	<LOQ	/	/
**Acid forms**					
**CBDA**	23,479	2,404	27,593	2,617	ns
**Δ^9^-THCA**	384	28	362	23	ns
**CBNA**	214	48	410	31	0.004
**CBCA**	457	33	<LOQ	/	0.008
**CBGA**	698	44	1,184	45	<0.001
**CBDVA**	2,862	276	1,233	56	<0.001
**Δ^9^- THCVA THCVA**	<LOQ	/	<LOQ	/	/

^a^ LOQ, limit of quantification 1 µg/g for all phytocannabinoids.

#### Un-Targeted Metabolomics: Phytocannabinoid Profiling and Putative Identification of Non-Phytocannabinoic Compounds by Means of HPLC-HRMS

**Figure 5:**
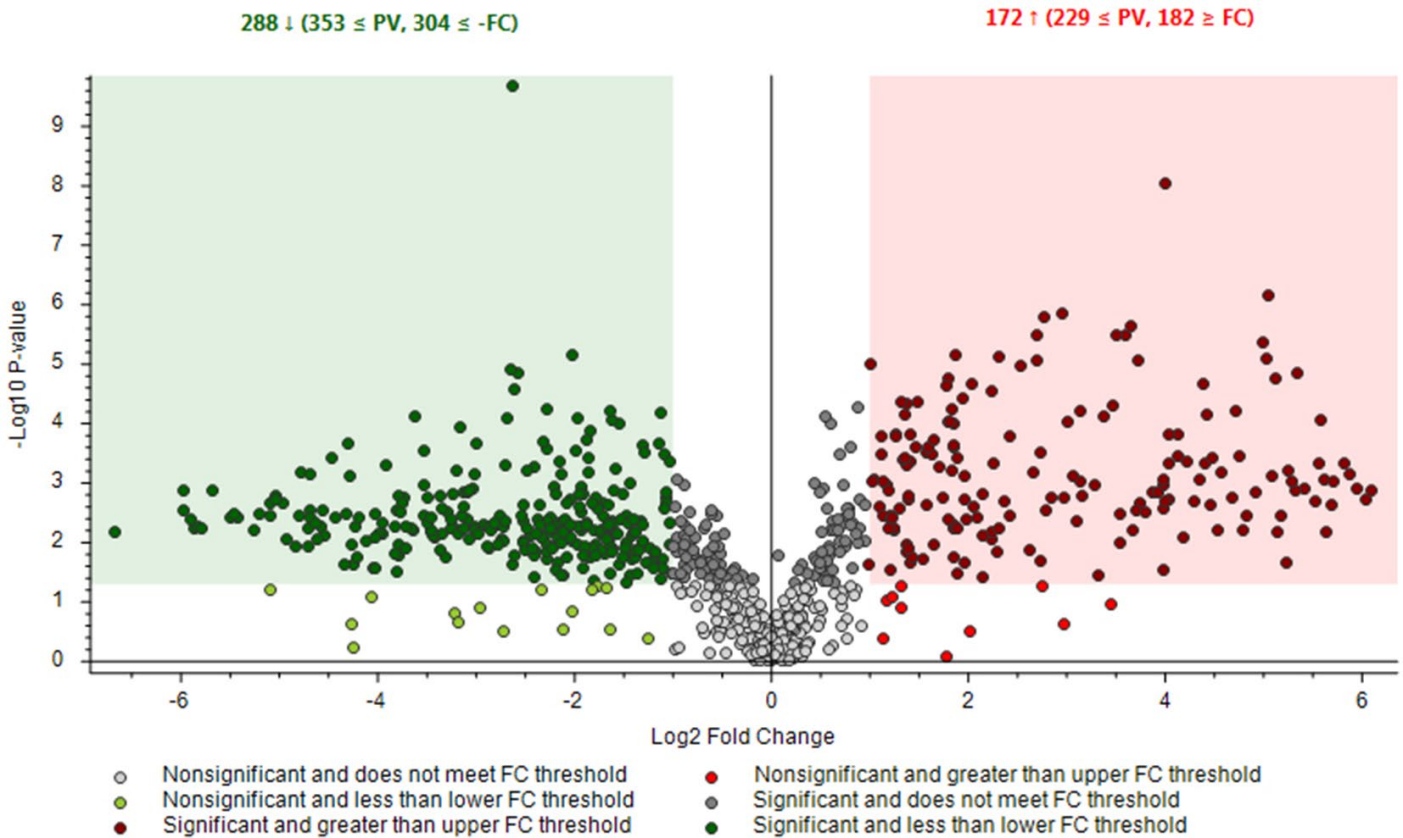
Differential analysis for the comparison between the relative intensity of chromatographic peak from Finola and Futura 75 samples. P-value (PV) was set on 0.05. Red region contains up-regulated signal, where the quantities from Futura were significantly higher than those found in Finola and were greater than the upper fold-change (FC) threshold. The green region comprises down-regulated peaks, where the quantity from Futura was significantly lower than that from Finola and was less than the lower FC threshold.

Two chemovars (each in five biological replicates) after being analyzed by LC-HRMS in FS-dd-MS^2^ acquisition mode were further processed by Compound Discoverer platform that enabled differential analysis applying Volcano plot model (**Figure 5**).

After performing differential analysis, the chromatographical signals were then subjected to the putative identification according to exact mass of the pseudomolecular ion (M+1)^+^ with appropriate mass fragmentation pattern found in the available databases (mzCloud, ChemSpider, FooDB) metabolomics platforms (MyCompoundID, Li et al., 2013) and/or reported in the literature (Brighenti et al., 2018; Calvi et al., 2018a; Berman et al., 2018; Citti et al., 2019). Our initial experiment data analysis involved about 100 known phytocannabionids that are listed in the recently published inventories (Hanus at al., 2016; Berman et al., 2018; ElSohly et al., 2017). The overall 43 additional phytocannabinoids from the seven phytocannabinoid subclasses were recognized in this study. In **Table 7**, the compounds that characterize the *Cannabis sativa* L. inflorescences have been by listed/identified by applying the CompoundDiscover platform. Both chemovars displayed a similar phytocannabinoids profile with some exceptions that are evident for the quantitative analysis presented above. The most abundant class was CBC-type with the C-1 and C-3 side chain length that was up-regulated in Finola, while the both neutral and acidic forms of C4 (Nor) and C-5 were found to be higher in Futura 75. The ample class of CBL-type (that derives from the CBC-counterparts) was also worth of noting, as the signals attributed to this class did not differ significantly between two varieties. The solid signals with the particular fragmentation of the cannabicitrans (CBCT-type) were observed in the both varieties without statistical significance. CBCT itself was identified according to the m/z cloud database spectrum, while other members of this group showed the analogous fragmentation, taking into account the structural differences concerning the side chain length and the carboxylic function for the acidic forms. A substantial, power signal designated the presence of cannabielsoinic acid, both C3 and C5 analogues, that are considered as main oxidation products of CBDVA and CBDA, respectively. Also, it is important to note the occurrence of the compounds that are produced by non-enzymatic, post-biosynthetical oxidative modifications of main phytocannabinoids: cannabifouranic acid, cannabicoumaronone, cannabichromanone, cannabiripsol, as well as acidic and neutral forms of 6,7-epoxycanabigerol.

**Table 7:** Putative identification of phytocannabinoids, flavonoids, lignans, stilbenoids, and alkaloids based on (full scan data dependent) FS-dda-MS^2^ characterization and chromatographical behavior.

Class	Compound	Formula	RT (min)	(M+H)^+^	dda-MS fragment^a^	FC, P-value Futura 75 vs./Finola^b^
***Phytocannabionoids***						
CBG cannabigerol -type	CBGVA	C_20_H_28_O_4_	26.03	333.2060	173.0962^a^	ns^c^
	6,7-epoxy-CBG	C_21_H_32_O_3_	24.94	333.2424	315.1867^a^	−3.41, 0.00045
	6,7-epoxy-CBGA	C_22_H_32_O_5_	23.25	377.2323	341.2113^a^	−1.97, 0.00022
	CBG	C_21_H_32_O_2_	27.26	317.2475	193.1223^a^	2.11, 0.000051
	CBGA	C_22_H_32_O_4_	27.21	361.2375	219.1017^a^	0.95, 0.00082
	Sesqui-CBG	C_26_H_40_O_2_	30.63	385.3173	193.1223^a^	ns
CBD (cannabidiol) –type	CBDO ^d^	C_17_H_22_O_2_	23.78	259.1693	187.0754^a^	ns
	CBDOA	C_18_H_22_O_4_	23.41	303.1591	285.1485^a^	ns
	CBDV	C_19_H_26_O_2_	25.33	287.2006	165.0914^a^	−4.97, 0.0037
	CBDVA	C_20_H_26_O_4_	24.94	331.1904	313.1801^a^	−3.28, 0.016
	Nor-CBD^e^	C_20_H_28_O_2_	26.36	301.2162	179.1070^a^	−2.85, 0.013
	Nor-CBDA	C_20_H_28_O_4_	25.85	345.2060	327.1956^a^	−3.22,0.0015
	CBD	C_21_H_30_O_2_	27.38	315.2319^a^	193.1223	ns
	CBDA	C_22_H_30_O_4_	26.77	359.2219	341.2114^a^	ns
Δ^9^-THC tetrahydrocannabinol –type	THCV	C_19_H_26_O_2_	27.17	287.2006	165.0914^a^	ns
	THCVA	C_20_H_26_O_4_	28.90	331.1904	313.1801^a^	ns
	Nor-THC	C_20_H_28_O_2_	28.34	301.2162^a^	179.1070	−1.09,0.0191
	Nor-THCA	C_20_H_28_O_4_	25.85	345.2060	327.1956^a^	−0.95, 0.00021
	THC	C_21_H_30_O_2_	29.43	315.2319^a^	193.1223	−2.91, 0.0052
	THCA	C_22_H_30_O_4_	30.54	359.2219	341.2114	ns
CBC cannabichromene -type	CBCO	C_17_H_22_O_2_	25.74	259.1693	187.0754^a^	−1.11, 0.025
	CBCOA	C_19_H_26_O_2_	27.65	303.1591	285.1485^a^	−0.58, 0.0042
	CBCV	C_19_H_26_O_2_	27.52	287.2006	165.0914^a^	−4.42, 0.0041
	CBCVA	C_20_H_26_O_4_	29.64	331.1904^a^	313.1801	−3.82, 0.0016
	Nor-CBC	C_20_H_28_O_2_	28.69	301.2162^a^	179.1070	0.72, 0.0078
	Nor-CBCA	C_20_H_28_O_4_	29.47	345.2060	327.1956^a^	4.0, 0.0001
	CBC	C_21_H_30_O_2_	29.82	315.2319	193.1223^a^	0.55, 0.0014
	CBCA	C_22_H_30_O_4_	31.00	359.2219	341.2114^a^	0.31, 0.0241
CBL cannabicyclol -type	CBLV	C_19_H_26_O_2_	28.58	287.2006^a^	165.0914	ns
	CBLVA	C_20_H_28_O_2_	29.47	331.2162	191.0703^a^	ns
	Nor-CBL	C_20_H_28_O_2_	29.62	301.2162	179.1070^a^	ns
	Nor- CBLA	C_20_H_28_O_4_	30.53	345.2060	205.0859^a^	ns
	CBL	C_21_H_30_O_2_	30.69	315.2319 ^a^	193.1223	ns
	CBLA	C_22_H_30_O_4_	33.34	359.2219	341.2114^a^	ns
CBCT cannabicitran -type	CBCTV	C_19_H_26_O_2_	28.58	287.2006^a^	165.0914	ns
	CBCT	C_21_H_30_O_2_	30.70	315.2319	193.1223^a^	ns
	CBCTA	C_22_H_30_O_4_	33.74	359.2219	341.2114^a^	ns
CBN cannabinol -type	CBN	C_21_H_26_O_2_	28.94	311.2007^a^	223.1118	−1.93, 0.0062
	CBNA	C_22_H_26_O_4_	30.03	355.1904	337.1800^a^	ns
CBE cannabielsoin -type	CBEVA	C_20_H_26_O_5_	23.06	347.1853	329.1755^a^	0.90, 0.0062
	CBEA	C_22_H_30_O_5_	26.55	375.2176	357.2061^a^	0.57, 0.0020
CBND cannabinodiol type	CBND	C_21_H_26_O_2_	23.62	311.2007^a^	223.1118	ns
	CBDNA	C_22_H_26_O_4_	23.34	355.1904	337.1800^a^	ns
Miscellaneous types	Cannabifuranic acid (CBFA)	C_22_H_26_O_4_	30.03	355.1904	337.1800^a^	ns
	Cannabiripsol (CBR)	C_21_H_32_O_4_	22.12	349.2373^a^	331.2276	ns
	Cannabicoumaronone (CBON)	C_21_H_28_O_3_	29.75	329.2111	98.9843^a^	ns
	Cannabichromanone (CNCN)	C_20_H_28_O_4_	25.46	333.2060	95.0857^a^	ns
	5-Acetoxy-6-geranyl-3-n-pentyl-1,4-benzoquinone	C_23_H_32_O_4_	28.81	373.2373	209.1173	1.7, 0.0074
***Flavonoids***						
Isoprenoid flavones	Cannaflavin A	C_26_H_28_O_6_	26.82	437.1964	313.0709^a^	4.14, 0.00037
	Cannaflavin B	C_21_H_20_O_6_	22.83	369.1333	313.0709^a^	2.7, 0.000017
	Cannaflavin C	C_26_H_28_O_6_	25.36	437.1964	313.0709	9.76, 0.000002
***Lignans***						
Phenolic amides	N-trans-coumaroyltyramine	C_17_H_17_NO_3_	15.46	284.1282	147.0442^a^	ns
	N-trans-feruloyltyramine	C_18_H_19_NO_4_	15.67	314.1387	177.0548	ns
	N-trans-caffeoyltyramine	C_17_H_17_NO_4_	13.39	300.1230	147.0442^a^	ns
Lignanamides	Cannabisin D	C_36_H_36_N_2_O_8_	18.31	625.2544	325.1072	1.68, 0.0004
	Grossamnide	C_36_H_38_N_2_O_9_	16.52	643.2650	462.1906	2.74, 0.021
***Stilbenoids***						
Phenathrenes	4,5-Dihydroxy-2,3,6-trimethoxy-9,10-dihydrophenanthrene	C_17_H_18_O_5_	20.34	303.1227	271.0964	−3.06, 0.023
	*Denbinobin*	C_16_H_12_O_5_	21.33	285.0575	242.0573	0.71, 0.0050
Dihydrostibenes	Canniprene	C_21_H_26_O_4_	23.40	343.1904	287.1270	−3, 0.0050
	Cannithrene 1	C_15_H_14_O_3_	18.45	243.1016	215.1067	4.15, 0.00016
	Cannithrene 2	C_16_H_16_O_3_	21.17	273.1124	241.0861^a^	1.37, 0.00075
	Cannabistilbene I	C_20_H_24_O_3_	24.94	313.1798	191.0720^a^	−0.66, 0.016
	Dihydroresveratrol	C_14_H_14_O_3_	25.68	231.1016	91.0544^a^	ns
Spiroindans	Cannabispiran	C_15_H_18_O_3_	19.00	247.1329	189.0909^a^	3.91, 0.0006
	Cannabispirenone	C_15_H_16_O_3_	18.29	245.1172^a^	163.0749	4.77, 0.00038
	Cannabispiradienone	C_15_H_14_O_3_	17.63	243.1016^a^	165.0702	4.15, 0.00015
	Cannabispiranol	C_15_H_20_O_3_	18.98	249.1485	137.0598^a^	4.65, 0.0008
***Alkaloids***						
	Hordenine	C_10_H_15_NO	1.54	166.1226^a^	121.0648	4.02, 0.000001

RT, retention time; (M+H)^+^, exact mass of pseudomolecular ion acquired in full scan mode; DDA-MS fragment ^a^ the base fragment in MS-MS spectrum; FC-fold change; Futura 75 vs./Finola ^b^ Positive value of FC indicates red region from Volcano plot graphic – the compounds that are up-regulated in Futura 75 vs. Finola, Negative value of FC indicates red region from Volcano plot graphic – the compounds that are down-regulated in Futura 75 vs. Finola; *ns* ^c^ not significant differences between two chemovars; *O* ^d^ (orcol) C1 side chain length for cannabinoids; *Nor* ^e^ C4 side chain length for cannabinoids.

**Figure 6:**
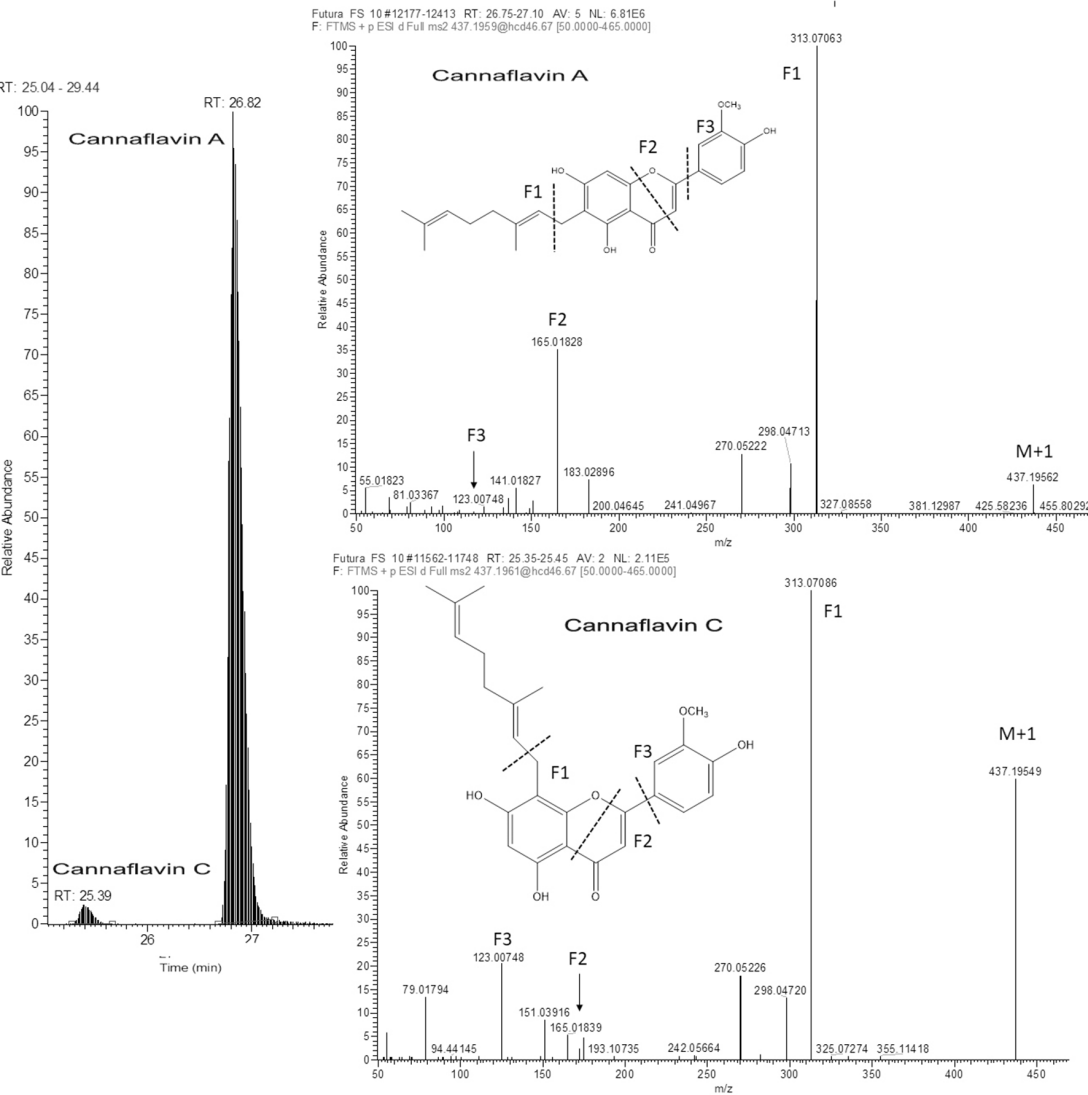
Extracted ion LC-HRMS chromatogram of Futura 75 inflorescence with the respective ful-MS^2^ spectra illustrating the presence of two isomeric compounds: Cannaflavin A and Cannaflavin C in Futura 75 inflorescence.

As far as non-phytocannabinoid secondary metabolites are concerned, untargeted metabolomic approach enabled the identification of the cannaflavines A, B, and C (**Table 7**). As it was not possible to find (neither in the literature nor in the available databases) their high resolution MS/MS spectrum done in positive mode, we have proposed the fragmentation outline as it was shown in the **Figure 6** for the cannaflavin A and C found in Futura 75. Those two isoprenic flavones are the isomeric compounds, displayed almost identical fragmentation pattern (with differences in relative abundance of pseudomolecular ion), but they can be distinguished very well by the chromatography. Position of isprenyl chain in the molecule cannaflavin A contributes to its higher lipophilicity compared to the cannaflavin C, which is more polar due to its compact structural aspect. Therefore, cannaflavin A had been eluted almost 2 min after the cannaflavin C. Futura 75 had been shown as much more affluent regarding all three cannaflavines (**Table 7**). The important issue is that cannaflavin C content was extremely high in Futura 75 than in Finola inflorescences where practically was present in traces.

Regarding the data obtained in positive ionization, it was possible to detect other secondary metabolites, already identified in some cannabis species (Pollastro et al., 2018). Between non-phytocannabinoid phenols, structurally unique compounds exist as lignans, spiroindan type, dihydrostilbene-type, dihydrophenantrene derivatives, stilbenoids, and cannabispirans. Lignans belong to two main groups: phenolic amides and lignanamides (Yan et al., 2015). Both varieties included in this study exhibited the analogous phenolic amides profile: the substantial amount of N-trans-coumaroyltyramine and N-trans-feruloyltyramine accompanied with approximately 10 times lower signal for the N-trans-caffeoyltyramine. N-trans-coumaroyltyramine and N-trans-feruloyltyramine found in *Cannabis sativa* L. are recognized as precursor of unique arylnapthalene bis-amides, cannabisins. The inflorescences of both genotypes contained substantial amount of cannabisin D and grossamide, while the presence of another 12 compounds of this class that was previously reported for the hemp seeds/fruits was not detected in our Futura 75 and Finola samples.

Stilbenoids identified in *Cannabis sativa* L. could be divided into three main structural types: phenanthrenes, dihydrostilbenes, and spiroindans. A rare quinoid stilbenoid denbinobin was well-defined in both chemovars, and intensity of its signals reveals that it is one of the major constituents of phenanthrene-type with Futura 75 as preponderate chemovar. This was accompanied with significant decline of 4,5-dihydroxy-2,3,6-trimethoxy-9,10-dihydrophenanthrene in Futura 75 compared to Finola, which implies toward divergent metabolic routes reserved for phenanthrenes. Furthermore, the differential analysis revealed that the content of dihydroresveratrol, which is considered as dyhydrostylbenes precursor, was unaffected, while the level of its derivates, cannithrenes, was significantly higher in Futura 75. The sub-class of cannbisilbenes was not significantly represented, apart cannabistilbene I that was featured for Finola. Noteworthy results emerged for the spiroindans: Futura 75 inflorescences were particularly reached in cannabispiran and its oxidation analouges, while their concentration in Futura turns out to be neglected. Alkaloids were not detected, with the exception of hordenine, predominately observed in Futura 75 samples. Anyway, here we have to underline that, bearing in mind alkaloids prominent polarity, the absence of this previously well-defined compounds belonging to this group may be attributed to the limitation of our chromatographic conditions to retain and reveal the polar nitrogen species.

#### Terpenoids Profile Evaluated by HS-SPME- GC-MS Methodology

The untargeted HPLC-HRMS approach has revealed also the presence of some terpenoids in the inflorescence exanimated herein, but due to its non-polar and high-volatile characteristics the HS-SPME-GC-MS analytical strategy remains the best methodology for comprehensive profiling of terpenoid fraction. Complete data concerning the terpenes profile are summarized and reported in **Table 8**. Overall, up to 83 volatile compounds composed the terpene fingerprint and are further divided in the classes. The sum of mono/di/tri terpenes did not vary significantly, but the two genotypes expressed qualitatively different profile: Finola inflorescences were characterized by higher quantities of β-ocimene and α-terpiolene, while in Futura 75 α- and β-pinene accompanied by extremely high β-myrcene were found as predominated. Both chemovars were partiality rich in sesquiterpenes: 45 different compounds from this subclass were identified, among which *trans*-caryophyllene and α-humulene were most abundant. The importance of ample sesquiterpene subclass is also reflected in the occurrence of sesqui-CBG that was documented during the HPLC-HRMS data elaboration (**Table 7**). The total content of sesquiterpenes was more than double in Finola than those discovered for Futura, while oxygenated products were more pronounced in Futura 75. Concretely, it should be noticed that fenchone and α-fenchyl alcohol were, from quantitative point of view, the most important oxygenated terpenes found. Although rather speculative, we have to indicate the possibility that α-fenchyl alcohol also exists in ester form coupled with CBD as we have recorded a strong signal that with the pseudomolecular ions (C_32_H_47_O_4_, 495.34689) and fragmentation pattern (CBDA structure) corresponds to the fenchyl-cannabidiolate.

**Table 8:** Terpenes extracted and identified by HS-SPME-GC/MS in Finola and Futura 75 inflorescences.

		FINOLA		FUTURA		Statistics
Rt^a^	*MONO/DI/TRI TERPENES*	Mean^b^	± SD	Mean	± SD	p-value^c^
5.17	α-Pinene	1,536.72	47.82	2,985.65	568.01	0.008
6.29	Cyclofeuchene	40.90	0.50	90.30	1.45	0.002
6.56	Camphene	39.72	9.76	99.55	18.19	<0.001
8.63	β-Pinene	546.61	44.02	1,272.66	179.66	<0.001
9.54	Sabinene	20.02	1.37	–	–	<0.001
10.96	δ-3-Carene	366.18	67.79	982.37	81.72	<0.001
11.91	α-Phellandrene	434.50	79.00	186.79	19.03	<0.001
12.66	β-Myrcene	5,809.47	241.91	7,834.47	770.81	0.008
12.76	α-Terpinene	241.02	30.49	124.20	11.98	<0.001
13.48	Limonene	360.68	34.53	1,261.26	126.56	<0.001
13.81	β-Phellandrene	788.01	57.45	524.45	21.63	<0.001
15.54	γ-Terpinene	566.42	49.09	197.97	13.38	<0.001
16.22	β-Ocimene	2,971.80	200.55	1,001.70	77.63	<0.001
16.46	p-Cymene	160.17	17.34	92.92	10.37	<0.001
17.16	α-Terpiolene	6,493.32	655.84	2,425.00	234.94	<0.001
18.62	(3E)-3-Icosene	26.09	6.38	–	–	<0.001
19.80	Alloocimene	13.60	1.72	4.10	0.54	<0.001
20.19	p-Mentha-1,5,8-triene	48.56	9.69	31.40	13.75	0.095
20.34	Neoalloocimene	46.58	7.29	15.92	2.88	0.008
21.33	Cymenene	130.67	14.86	77.84	16.46	<0.001
21.65	(2-Methylprop-1-enyl)-cyclohexa-1,5-diene	41.62	1.48	–	–	<0.001
	**Total**	**20,765.66**		**19,208.54**		
	***SESQUITERPENES***					
22.07	α-Cubebene	26.71	4.05	–	–	<0.001
22.47	α-Ylangene	28.57	4.34	26.27	6.37	ns
22.86	α-Copaene	19.64	5.68	6.90	1.99	0.008
24.07	Zingiberene	–	–	17.44	4.27	<0.001
24.61	Sesquiterpene	189.60	48.99	119.36	27.05	0.008
24.60	Sesquiterpene	–	–	134.59	30.50	<0.001
24.90	α-Bergamotene	–	–	323.74	34.84	<0.001
25.22	Trans-Caryophyllene	5,003.84	981.42	1,126.71	194.40	<0.001
25.40	Sesquiterpene	65.84	11.91	–	–	<0.001
25.71	Sesquiterpene	23.83	6.58	–	–	<0.001
25.85	Sesquiterpene	5.16	2.76	–	–	<0.001
25.73	β-Santalene	15.17	6.72	26.91	6.79	ns
25.94	α-Gurjunene	27.84	11.27	19.94	5.82	ns
26.02	Aromadendrene	14.53	6.03	32.53	9.49	0.007
26.10	Sesquiterpene	21.01	4.44	–	–	<0.001
26.32	Sesquiterpene	5.08	1.15	–	–	<0.001
26.53	α-Humulene	1,537.92	379.28	523.43	78.85	<0.001
26.60	γ-Selinene	193.50	44.13	0.46	0.09	<0.001
26.64	β-Farnesene	–	–	356.87	87.56	<0.001
26.90	Sesquiterpene	–	–	66.53	20.25	<0.001
26.95	α-Cadinene	101.11	35.10	15.23	7.38	<0.001
27.34	δ-Guaiene	43.68	44.14	–	–	<0.001
27.44	β-Selinene	240.43	62.10	201.57	52.05	ns
27.57	α-Selinene	148.40	40.01	139.34	43.06	ns
27.77	β-Bisabolene	113.82	41.26	111.58	38.00	ns
27.95	Sesquiterpene	1.95	0.77	–	–	<0.001
28.03	Sesquiterpene	4.42	2.17	–	–	<0.001
28.09	α-Guaiene	37.35	15.61	22.55	7.40	ns
28.23	α-Farnesene	60.81	32.72	44.74	18.39	ns
28.29	α-Cadinene	25.45	14.60	17.89	6.05	ns
28.48	β-Maaliene	93.13	34.36	94.72	32.58	ns
28.55	β-Sesquiphellandrene	11.82	4.81	16.80	5.72	ns
28.64	Selina-3.7(11)-diene	120.23	19.38	240.13	56.96	0.008
28.92	α-Muurolen	1.34	0.39	–	–	<0.001
29.00	Eremophilene	–	–	3.29	1.18	<0.001
29.03	(+)-Sativene	147.80	49.89	–	–	<0.001
29.16	Sesquiterpene	18.20	7.38	8.61	3.86	< 0.001
29.51	Sesquiterpene	48.23	19.96	78.02	29.49	ns
29.61	Calamenene	2.86	0.74	–	–	0.001
32.30	Santalol	1.25	0.75	–	–	0.001
32.66	Nerolidol	2.11	0.76	2.65	0.78	ns
33.51	Isolongifolen	1.58	0.46	–	–	<0.001
33.80	Sesquiterpene	2.59	1.31	2.02	0.38	ns
34.36	α-Bisabolol	2.45	1.20	–	–	<0.001
	**total**	**8,409.25**		**3,780.79**		
	***OXIGENATED TERPENES***					
20.03	Fenchone	–	–	102.13	10.52	<0.001
23.89	Cis-sabinene hydrate	17.96	7.77			0.008
22.13	Linalool oxide	1.44	0.86	29.22	3.81	<0.001
22.00	Trans-3-caren-2-ol	109.70	15.61	35.67	5.30	<0.001
23.02	β-Pinone	0.93	0.17	–	–	0.001
24.00	Pinanol	–	–	48.46	25.57	<0.001
24.18	3-Pinanone	–	–	14.71	3.36	<0.001
24.20	β-Linalool	18.81	3.89	17.98	4.11	0.750
24.78	α-Fenchyl alcohol	–	–	73.25	11.31	<0.001
25.28	4-Terpineol	8.30	3.68	15.59	4.68	0.056
26.23	Trans-Pinocarveol	10.15	2.84	–	–	<0.001
27.08	α-Terpineol	31.27	13.64	5.08	2.46	<0.001
29.35	3-Terpinolenone	6.14	2.37	–	–	<0.001
26.79	Carotol	8.01	2.66	–	–	<0.001
31.93	Caryophyllene oxide	32.47	17.24	13.10	4.65	0.016
32.13	Alloaromadendroneoxid	0.73	0.33	–	–	0.008
32.55	Humulene oxide	6.14	3.50	4.94	2.20	0.536
34.58	Eugenol	2.36	0.18	–	–	0.008
	**Total**	**254.41**		**380.13**		

Experimental conditions as in Sections material and methods.

RT^a^, retention time (min); Mean^b^, Data are given as mean *±* SD, n = 5, independent biological replicates); p-value^c^, T-test with 95% two-tailed confidence interval for difference of means; Bold, values are referred to the main constituents of the analyzed samples.

## Discussion

To support a sustainable development of industrial hemp in mountain areas, proper agronomic techniques should be optimized to preserve inflorescence and/or seed quality during the plant seasoning. This would enable suitable industrial processing and would improve high-added value applications, especially for the nutraceutical and homoeopathic purposes (Calzolari et al., 2017). Hemp has a wide range of environmental adaptation, but varieties tend to perform better in their instinctive areas of growth (Dempsey, 1975) what was confirmed by our results regarding the functional strategy. Particularly, Finola has been proven as more stress-tolerant variety in comparison with Futura 75. These data contribute to fill the lack of studies of intraspecific variation of CSR strategy; in fact, until now, only May et al. (2017), Giupponi and Giorgi 2019a; Giupponi and Giorgi et al., 2019b, and Giupponi et al., 2017; Giupponi et al., 2019 have studied this intraspecific variation in *Arabidopsis thaliana*, *Linaria tonzigii*, and *Fagopyrum tataricum*, respectively. Finola is a Finnish short-cycle variety, considered as a self-flourishing for its ability to bloom in about 3 months and with a high germinability (Baldini et al., 2018). Having been conceived for northern Europe, it remains short and therefore more manageable, more “compact,” and more stress tolerant. Futura as monoic French variety of medium height (2–3 mt) with intermediate flowering is suitable for fabric transformation and with discrete levels of seed production as a main product.

Nowadays, environmental concerns and multi-purpose production have brought renewed interest in industrial hemp; however, there is little information that concerns phytochemical composition to support hemp cultivation in mountain areas. Besides hemp fiber production, there is growing interest in cultivating industrial hemp for other purposes such as using its inﬂorescence for extracting essential oils and its seeds for alimentary oil and ﬂour production (Campiglia et al., 2017). However, few studies have compared the performance of the current commercial chemovar of industrial hemp (Tang et al., 2016) and there is little available information regarding agronomical practices. Therefore, considering the lack of information on hemp genotypes, it is difficult for mountain farmers to select the most suitable genotype for different kinds of utilization.

The result that regards qualitative analysis of inflorescence phytocannabinoids profile in the inflorescences points out that, under same environmental conditions that yield the major cannabinoid, CBDA is similar in both varieties. The difference that is significant regarding the neutral derivate CBD indicated the higher amount revealed in Finola could be indirectly caused by genetic predisposition of Finola to flower in the full summer season when the average daily temperatures are higher than those that are present when Futura 75 develops its inflorescences (late summer/early autumn). Therefore, flowering season, itself, may have led to a partial conversion of the parent CBDA into its neutral counterpart, *via* a decarboxylation process, which naturally occurs under the action of heat and light. The sum of problematic, potentially toxic THC and THCA was found to be low (under 0.1%), which confirms the possibility to use inflorescences in nutraceuticals purposes. Notable amount of neutral CBDV for Finola opens possibility to exploit this variety for the medicinal (galenic) preparation as CBDV is considered to express a beneficial effect on human health (Iannotti et al., 2014).

Apart from the 14 phytocannabinoids that were quantified, untargeted metabolomics approached offered in-depth recognitions of the possible dissimilarities in the chemical profile between two chemovars. For the cannabinoids fraction, apart for the evident alterations that are produced by genetic differences (alterations found for CBC class, for example), it is important to consider also the phytocannabionoids that are produced due to non-enzymatic modifications of main phytocannabionids. In the first place, there is a substantial power signal that designates the presence of cannabielsoinic acid, both C3 and C5 analogues, that are considered as main oxidation products of CBDVA and CBDA, respectively. Also, the presence of four compounds from the miscellaneous type indicates that cultivation under uniform, mountain growing environment has led to the biotransformation of main phytocannabionids to the particular oxidation forms that were revealed as cannabifouranic acid (CBDFA) that derives from the CBDA, and the cannabicoumaronone and cannabichromanone originating from THC. Also, the appearance of cannabiripsol that is specific glycolic forms of THC indicates toward prompt hydroxylation of mayor phytocannabinoids. The presence of the 6,7-epoxycanabigerol acidic and neutral form with significantly higher amount in the Finola inflorescence should not be undervalued as it is not still clear weather the epoxidation had occurred as the part of the early phytocannabinoids metabolic pathway during the flowering, or it had aroused as result of the post-harvest CBG oxidative modifications.

Cannflavins, which belong to the class of prenylflavonoids, are secondary metabolite exclusive for the *Cannabis* genus and were detected in both varieties. Their notable presence points towards metabolic pathway at the outgoings of the polyketide cannabinoid machinery (Calzolari et al., 2017). The notably higher level of cannaflavin C detected in Futura 75 could be a consequence of the lower average temperature combined with high solar radiation experienced at the beginning of plant flowering. In fact, it was reported that different classes of flavonoids are involved in plant protection mechanisms, specifically for their radical scavenger activity and screening ability against short wavelength UV-B light (Agati and Tattini, 2010). Furthermore, Futura 75 inflorescences was characteristically rich in cannabispiran and its oxidation analogues, which can be in relation with the fact that Futura 75 is more susceptible to oxidative stress.

Considering the terpenoids fraction, our results are qualitatively comparable with those reported by others (Bertoli et al., 2010; Elzinga et al., 2015; Aizpurua-Olaizola et al., 2016; Benelli et al., 2018, Namdar et al., 2018). The particularity of both chemovars presented in this study is the presence of the strong signal of sesqui-CBG accompanied with very reach sesquiterpenes fraction, indicating that geographic origin accompanied with environmental conditions is one of the important variables that determine the terpene fingerprint (Giorgi et al., 2013b; Marchini et al., 2014; Giupponi et al., 2017). The presumed appearance of fenchyl-cannabidiolate might also be defined as possible discrimination factor that concerns cultivation/ecological conditions. Apart from environmental impact, a genetic predisposition of variety could thoughtfully affect the terpene family: the qualitative profile of our Futura inflorescence is similar to this recently reported by Benelli et al. (2018). The significant differences found in the quali-quantitative terpenes profile (such as α- and β-pinene, myrcene, terpinolene, β-ocimene, *trans*-caryophyllene, and α-humulene) between Futura and Finola inflorescences reported herein permit to distinguish monoecious from dioecious hemp cultivars (Bertoli et al., 2010).

The fatty acid composition is a primary value of hemp seed. The composition of the fatty acid profile has been demonstrated to vary according to the plant cultivars. Both varieties grown in mountain have been shown as a good resource of PUFA (71.98% for Finola and 77.61% for Futura 75). Fundamentally, ω6/ω3 is found to be in range 2.5–3:1 (Simopoulos, 2008), which has been claimed as ideal for human nutrition. In fact, the value of 3.21 calculated for Futura 75 qualifies this genotype as favorable for human diet (Callaway, 2004, Galasso et al., 2016), Particularly, the level of ω6 is thus an interesting resource for manufacturing dietary supplement (Mölleken and Theimer, 1997). Finola contained much lower amount of ω3, which influenced final ω6/ω3 (6.25), which is higher than values reported recently (Galasso et al., 2016) for Finola and for the other varieties of industrial hemp (Petrović et al., 2015). The principal difference in ω3 PUFA profile involves the linolenic acid, which was surprisingly two times lower in Finola than in Futura 75. On the other hand, our findings have demonstrated that Finola has an important content of GLA if grown in an mountain area of the Alps, which is in accordance with results published by Mölleken and Theimer (1997) that the cultivars from the cooler regions displaying a higher concentration of GLA (Callaway, 2004; Vonapartis et al., 2015).

Our results confirmed that about 65% of the total hemp protein consists of a single storage protein, edestin, a hexamer composed of acidic and basic subunits linked by disulfide bonds. It has been stated that, differently from soybean seeds that are abundant in trypsin inhibitors that require thermal treatment for inactivation, hemp seeds present very low amounts of these anti-nutritional factors and are therefore more digestible (Malomo et al., 2015). Due to the high content in essential amino acids of edestin, hemp seed proteins could be considered as a superfood, possessing a health-promoting property that might reduce a risk of disease or improving some aspect of well-being.

## Conclusions

Hemp is recognized as a crop that could be cultivated in different surroundings, exploiting marginal land. Generally, it can be grown without pesticides (Desanlis et al., 2013) and with a low input technique (Struik et al., 2000), so it becomes a very interesting option for farm working in organic regime in very delicate environments as mountain areas. That is reason why it is considered as alternative viable crop for sustainable agriculture (Amaducci et al., 2015). As a multifunctional crop, it can have different end use: traditional ones as fiber but also innovative ones as the use of seeds and inflorescences as sources of interesting bioactive secondary metabolites for nutraceutical (Frassinetti et al., 2018), medicinal (Calvi et al., 2018b), and cosmetic (Sapino et al., 2005) purposes or for producing essential oils as natural flavor and fragrance additives (Bertoli et al., 2010). Comprehensive quality study of two fiber-type hemp varieties cultivated in Italian Alps points out a specific, legal, and safe cannabinoids profile and particular terpene composition in the inflorescences that is followed by a favorable polyunsaturated fatty acids and protein content in the seeds.

Due to its different application and since this crop was abandoned for long time in mountain environment, geographical provenience of hemp should be considered in selecting a variety that would be suitable for a specific nutraceutical destination. Further and targeted studies should be addressed to the ecological and phytochemical behavio of most popular varieties in diverse edaphic and environmental conditions. This would be important for the possible end-use of the hemp and to support mountain farmers to select the correct variety for their purposes and agronomic environment. Additionally, due to its ecological and phytochemical complexity, the comprehensive chemical profiling of this plant could be an interesting case study for investigations on plant physiology and plant behavior in response to different biotic and abiotic environmental factors.

## Data Availability Statement

The raw data supporting the conclusions of this manuscript will be made available by the authors, without undue reservation, to any qualified researcher.

## Author Contributions

AG, RP, VL, SP, LG, and MC conceived and designed the study and interpreted the results. RP, VL, MC, CCi, and CCa performed phytochemical analyses and statistical analyses. VL and LG followed the experimental fields and performed ecological and statistical analyses. RP and CCi completed metabolomic examination. All the authors contributed to writing, with AG as the leading author.

## Funding

This research was supported by the “FISR-MIUR Italian Mountain Lab” project.

## Conflict of Interest

The authors declare that the research was conducted in the absence of any commercial or financial relationships that could be construed as a potential conflict of interest.

## References

[B1] Agati G. Tattini M. (2010). Multiple functional roles of flavonoids in photoprotection. New Phytol. 186, 786–793. 10.1111/j.1469-8137.2010.03269.x 20569414

[B2] Aizpurua-Olaizola O. Soydaner U. Öztürk E. Schibano D. Simsir Y. Navarro P. (2016). Evolution of the cannabinoid and terpene content during the growth of *Cannabis sativa* plants from different chemotypes. J. Nat. Prod. 79, 324–331. 10.1021/acs.jnatprod.5b00949 26836472

[B3] Amaducci D. Scordia F. H. Liu Q. Zhang H. Guo G. Testa S. L. (2015). Key cultivation techniques for hemp in Europe and China. Ind. Crops Prod. 68, 2–16. 10.1016/j.indcrop.2014.06.041

[B4] Baldini M. Ferfuia C. Piani B. Sepulcri A. Dorigo G. Zuliani F. (2018). The performance and potentiality of monoecious hemp (*Cannabis sativa* L.) cultivars as a multipurpose crop. Agronomy 162, 1–16. 10.3390/agronomy8090162

[B5] Benelli G. Pavela R. Lupidi G. Nabissi M. Petrelli R. Ngahang Kamte S. L. (2018). The crop-residue of fiber hemp cv. Futura 75: from a waste product to a source of botanical insecticides. Environ. Sci. Pollut. Res. Int. 25 (11), 10515–10525. 10.1007/s11356-017-0635-5 29105041

[B6] Berman P. Futoran K. Lewitus G. M. Mukha D. Benami M. Shlomi T. (2018). A new ESI-LC/MS approach for comprehensive metabolic profiling of phytocannabinoids in Cannabis. Sci. Rep. 8, 14280. 10.1038/s41598-018-32651-4 30250104 PMC6155167

[B7] Bertoli A. Tozzi S. Pistelli L. Angelini L. G. (2010). Fibre hemp inflorescences: from crop-residues to essential oil production. Ind. Crops Prod. 32, 329–337. 10.1016/j.indcrop.2010.05.012

[B8] Blasi C. Capotorti G. Copiz R. Guida D. Mollo B. Smiraglia D. (2014). Classification and mapping of the ecoregions of Italy. Plant Biosyst. 148, 1255–1345. 10.1080/11263504.2014.985756

[B9] Bligh E. G. Dyer W. J. (1959). A rapid method of total lipid extraction and purification. Can. J. Biochem. Physiol. 37, 911–917. 10.1139/y59-099 13671378

[B10] Bonini S. A. Premoli M. Tambaro S. Kumar A. Maccarinelli G. Memo M. (2018). *Cannabis sativa*: a comprehensive ethnopharmacological review of a medicinal plant with a long history. J. Ethnopharmacol. 227, 300–315. 10.1016/j.jep.2018.09.004 30205181

[B11] Bradford M. M. (1976). A rapid and sensitive method for the quantitation of microgram quantities of protein utilizing the principle of protein-dye binding. Anal. Biochem. 72, 248–254. 10.1016/0003-2697(76)90527-3 942051

[B12] Brighenti V. Pellati F. Steinbach M. Maran D. Benvenuti S. (2018). Development of a new extraction technique and HPLC method for the analysis of non-psychoactive cannabinoids in fibre-type Cannabis sativa L. (hemp). J. Pharm. Biomed. Anal. 143, 228–236. 10.1016/j.jpba.2017.05.049 28609672

[B13] Callaway J. C. (2004). Hempseed as a nutritional resource: an overview. Euphytica 140, 65–72. 10.1007/s10681-004-4811-6

[B14] Calvi L. Pavlovic R. Panseri S. Giupponi L. Leoni V. Giorgi A. (2018a). “Quality traits of Medical *Cannabis sativa* L. inflorescences and derived products based on comprehensive analytical investigation,” in Recent Advances in Cannabinoid Research. (London: IntechOpen Limited) 10.5772/intechopen.79539

[B15] Calvi L. Pentimalli D. Panseri S. Giupponi L. Gelmini F. Beretta G. (2018b). Comprehensive quality evaluation of medical *Cannabis sativa* L. inflorescence and macerated oils based on HS-SPME coupled to GC–MS and LC-HRMS (q-exactive orbitrap^®^) approach. J. Pharm. Biomed. Anal. 150, 208–219. 10.1016/j.jpba.2017.11.073 29247961

[B16] Calzolari D. Magagnini G. Lucini L. Grassi G. Appendino G. B. Amaducci S. (2017). High added-value compounds from *Cannabis* threshing residues. Ind. Crops Prod. 108, 558–563. 10.1016/j.indcrop.2017.06.063

[B17] Campiglia E. Radicetti E. Mancinelli R. (2017). Plant density and nitrogen fertilization affect agronomic performance of industrial hemp (*Cannabis sativa* L.) in Mediterranean environment. Ind. Crops Prod. 100, 246–254. 10.1016/j.indcrop.2017.02.022

[B18] Citti C. Linciano P. Panseri S. Vezzalini F. Forni F. Vandelli M. A. (2019). Cannabinoid profiling of hemp seed oil by liquid chromatography coupled to high-resolution mass spectrometry. Front. Plant Sci. 10, 120. 10.3389/fpls.2019.00120 30815007 PMC6381057

[B19] Dempsey J. M. (1975). Hemp fibre crops. Gainesville, FL: University of Florida Press, 46–89.

[B20] Desanlis F. Cerruti N. Warner P. (2013). Hemp agronomics and cultivation. Ed. Bouloc p. Wollingford, UK: CABI, 98–124. 10.1079/9781845937935.0098

[B21] Elzinga S. Fischedick R. Podkolinski J. Raber C. (2015). Cannabinoids and terpenes as chemotaxonomic markers in cannabis. Nat. Prod. Chem. Res. 3, 81. 10.4172/2329-6836.1000181

[B22] ElSohly M. Radwan M. Gul W. Chandra S. Galal A. (2017). “Phytochemistry of Cannabis sativa,” in Phytocannabinoids: progress in the chemistry of organic natural products, vol. 103 . Eds. Kinghorn A. D. Falk H. Gibbons S. Kobayashi J. (Switzerland: Springer International Publishing), 1–36. 10.1007/978-3-319-45541-9_1 28120229

[B23] EU Regulation (2013). No 1307/2013 of the European Parliament and of the Council of 17 December 2013 establishing rules for direct payments to farmers under support schemes within the framework of the common agricultural policy and repealing Council Regulation (EC) No 637/2008 and Council Regulation (EC) No 73/2009. Off. J. Eur. Union, 347/608.

[B24] Faux A. M. Draye X. Lambert R. Andrimont R. Raulier P. Bertin P. (2013). The relationship of stem and seed yields to flowering phenology and sex expression in monoecious hemp (*Cannabis sativa* L.). Eur. J. Agron. 47, 11–22. 10.1016/j.eja.2013.01.006

[B25] Frassinetti S. Moccia E. Caltavuturo L. Gabriele M. Longo V. Bellani L. (2018). Nutraceutical potential of hemp (*Cannabis sativa* L.) seeds and sprouts. Food Chem. 262, 56–66. 10.1016/j.foodchem.2018.04.078 29751921

[B26] Galasso I. Russo R. Mapelli S. Ponzoni E. Brambilla I. M. Battelli G. (2016). Variability in seed traits in a collection of *Cannabis sativa* L. Genotypes. Front. Plant Sci. 7, 688. 10.3389/fpls.2016.00688 27242881 PMC4873519

[B27] Giorgi A. Panseri S. Nanayakkara N. N. M. C. Chiesa L. M. (2012). HS-SPME-GC/MS analysis of the volatile compounds of *Achillea collina*: evaluation of the emissions fingerprint induced by *Myzus persicae* infestation. J. Plant Biol. 55 (3), 251–260. 10.1007/s12374-011-0356-0

[B28] Giorgi A. Panseri S. Mattara M. S. Andreis C. Chiesa L. M. (2013a). Secondary metabolites and antioxidant capacities of Waldheimia glabra (decne.) regel from Nepal. J. Sci. Food Agric. 93 (5), 1026–1034. 10.1002/jsfa.5839 22903742

[B29] Giorgi A. De Marinis P. Granelli G. Chiesa L. M. Panseri S. (2013b). Secondary metabolite profile, antioxidant capacity, and mosquito repellent activity of *Bixa orellana* from Brazilian Amazon region. J. Chem. 409826. 10.1155/2013/409826

[1702] Giorgi A. Manzo A. Nanayakkara N. N. Giupponi L. Cocucci M. Panseri S. (2015). Effect of biotic and abiotic stresses on volatile emission of *Achillea collina* Becker ex Rchb. Natural Product Research 29, 1695– 10.1080/14786419.2014.997725 25564988

[B31] Giupponi L. Borgonovo G. Panseri S. Giorgi A. (2019). Multidisciplinary study of a little known landrace of Fagopyrum tataricum Gaertn. of Valtellina (Italian Alps). Genet. Resour. Crop Evol. 66, 783–796. 10.1007/s10722-019-00755-z

[B32] Giupponi L. Giorgi A. (2019a). A contribution to the knowledge of Linaria tonzigii Lona, a steno-endemic species of the Orobie Bergamasche Regional Park (Italian Alps). Eco. Mon. 11, 16–23. 10.1553/eco.mont-11-1s16

[B33] Giupponi L. Giorgi A. (2019b). Effectiveness of modern leaf analysis tools for the morpho-ecological study of plants: the case of *Primula albenensis* Banfi et Ferl. Nor. J. Bot. 37, e02386, 1–10 10.1111/njb.02386

[B34] Giupponi L. Pentimalli D. Manzo A. Panseri S. Giorgi A. (2017). Effectiveness of fine root fingerprinting as a tool to identify plants of the Alps: results of preliminary study. Plant Biosyst. 152, 464–473. 10.1080/11263504.2017.1306003

[B35] Grime J. P. (1974). Vegetation classification by reference to strategies. Nature 250, 26–31. 10.1038/250026a0

[B36] Grime J. P. (1977). Evidence for the existence of three primary strategies in plants and its relevance to ecological and evolutionary theory. Am. Nat. 111, 1169–1194. 10.1086/283244

[B37] Grime J. P. (2001). Plant strategies, vegetation processes and ecosystem properties. Chichester: Wiley & Sons.

[B38] Hanuš L. O. Meyer S. M. Muñoz E. Taglialatela-Scafati O. Appendino G. (2016). Phytocannabinoids: a unified critical inventory. Nat. Prod. Rep. 33, 1357–1392. 10.1039/C6NP00074F 27722705

[B39] Hillig K. W. (2004). A chemotaxonomic analysis of terpenoid variation in *Cannabis*. Biochem. Syst. Ecol. 32, 875–891. 10.1016/j.bse.2004.04.004

[B40] Iannotti F. A. Hill C. L. Leo A. Alhusaini A. Soubrane C. Mazzarella E. (2014). Nonpsychotropic plant cannabinoids, cannabidivarin (CBDV) and cannabidiol (CBD), activate and desensitize transient receptor potential vanilloid 1 (TRPV1) channels *in vitro*: potential for the treatment of neuronal hyperexcitability. ACS Chem. Neurosci. 5, 1131–1141. 10.1021/cn5000524 25029033

[B41] Legge (242/2016) Disposizioni per la promozione della coltivazione e della filiera agroindustriale della canapa. (16G00258) 2016 https://www.gazzettaufficiale.it/eli/gu/2016/12/30/304/sg/pdf.

[B42] Lewis M. A. Russo E. B. Smith K. M. (2018). Pharmacological foundations of cannabis chemovars. Planta Med. 84, 225–233. 10.1055/s-0043-122240 29161743

[B43] Li L. Li R. Zhou J. Zuniga A. Stanislaus A. E. Wu Y. (2013). MyCompoundID: using an evidence-based metabolome library for metabolite identification. Anal. Chem. 85, 3401–3408. 10.1021/ac400099b 23373753

[B44] Marchini L. M. Charvoz C. Dujourdy L. Baldovini N. Filippi J. J. (2014). Multidimensional analysis of cannabis volatile constituents: identification of 5,5-dimethyl-1-vinylbicyclo[2.1.1] hexane as a volatile marker of hashish, the resin of Cannabis sativa. J. Chromatogr. A. 1370, 200–215. 10.1016/j.chroma.2014.10.045 25454145

[B45] Malomo S. A. Onuh J. O. Girgih A. T. Aluko R. E. (2015). Structural and antihypertensive properties of enzymatic hemp seed protein hydrolysates. Nutrients 7, 7616–7632. 10.3390/nu7095358 26378569 PMC4586553

[B46] May R. L. Warner S. Wingler A. (2017). Classification of intra-specific variation in plant functional strategies reveals adaptation to climate. Ann. Bot. 119, 1343–1352. 10.1093/aob/mcx031 28369157 PMC5604582

[B47] Mamone G. Picariello G. Ramondo A. Nicolai M. A. Ferranti P. (2019). Production, digestibility and allergenicity of hemp (*Cannabis sativa* L.) protein isolates. Food Res. Int. 115, 562–571. 10.1016/j.foodres.2018.09.017 30599980

[B48] McPartland J. M. (2018). Cannabis systematics at the levels of family, genus, and species. Cannabis Cannabinoid Res. 3, 1. 10.1089/can.2018.0039 30426073 PMC6225593

[B49] Mediavilla V. Jonquera M. Schmid-Slembrouck I. Soldati A. (1998). Decimal code for growth stages of hemp (*Cannabis sativa* L.). J. Int. Hemp Assoc. 5, 68–74.

[B50] Metcalf L. C. Shmitz A. A. Pelka J. R. (1996). Rapid preparation of methyl esters from lipid for gas chromatography analysis. Anal. Chem. 38, 514–515. 10.1021/ac60235a044

[B51] Mölleken H. Theimer R. R. (1997). Survey of minor fatty acids in Cannabis sativa L. fruits of various origins. J. Int. Hemp Assoc. 4, 13–17.

[B52] Namdar D. Moran M. Ion A. Koltai H. (2018). Variation in the compositions of cannabinoid and terpenoids in *Cannabis sativa* derived from inflorescence position along the stem and extraction methods. Ind. Crops Prod. 113, 376–382. 10.1016/j.indcrop.2018.01.060

[B53] Pavlovic R. Nenna G. Calvi L. Panseri S. Borgonovo G. Giupponi L. (2018). Quality traits of “cannabidiol oils”: cannabinoids content, terpene fingerprint and oxidation stability of european commercially available preparations. Molecules 23, 1920. 10.3390/molecules23051230 PMC6100014 29783790

[B54] Petrović M. Debeljak Ž. Kezić N. Džidara P. (2015). Relationship between cannabinoids content and composition of fatty acids in hempseed oils. Food Chem. 170, 218–225. 10.1016/j.foodchem.2014.08.039 25306338

[B55] Pierce S. Negreiros D. Cerabolini B. E. L. Kattge J. Dìaz S. Kleyer M. et al. (2017). A global method for calculating plant CSR ecological strategies applied across biomes world-wide. Funct. Ecol. 31, 444–457. 10.1111/1365-2435.12722

[B56] Pollastro F. Minassi A. Fresu L. G. (2018). Cannabis Phenolics and their Bioactivities. Curr. Med. Chem. 25 (10), 1160–1185. 10.2174/0929867324666170810164636 28799497

[B57] Plant Variety Catalogues, Databases & Information Systems. (1995). Available online: https://ec.europa.eu/food/plant/plant_propagation_material/plant_variety_catalogues_databases_en [Accessed January 18, 2019].

[B58] R Development Core Team (2018). R: a language and environment for statistical computing. Vienna, Austria: R Foundation for Statistical Computing http://www.r-project.org.

[B59] Rivas-Martinez S. Rivas-Saenz S. (2009). Sistema de Clasificacion Bioclimatica Mundial. Espana: Centro de Investigaciones Fitosociologicas http://www.globalbioclimatics.org.

[B60] Russo E. B. (2019). The case for the entourage effect and conventional breeding of clinical cannabis: no “strain,” no gain. Front. Plant Sci. 9, 1969. 10.3389/fpls.2018.01969 30687364 PMC6334252

[B61] Russo E. B. (2011). Taming THC: potential cannabis synergy and phytocannabinoid-terpenoid entourage effects. Br. J. Pharmacol. 163, 1344–1364. 10.1111/j.1476-5381.2011.01238.x 21749363 PMC3165946

[B62] Salentijn E. M. J. Zhang Q. Amaducci S. Yang M. Trindade L. (2015). New developments in fiber hemp (*Cannabis sativa* L.) breeding. Ind. Crops Prod. 68, 32–41. 10.1016/j.indcrop.2014.08.011

[B63] Sapino S. Carlotti M. E. Peira E. Gallarate M. (2005). Hemp-seed and olive oils: their stability against oxidation and use in O/W emulsions. Int. J. Cosmet. Sci. 27, 355–356. 10.1111/j.1467-2494.2005.00290_2.x 16130045

[B64] Spertino S. Cipriani V. De Angelis C. Giuffrida M. G. Marsano F. Cavaletto M. (2012). Proteome profile and biological activity of caprine, bovine and human milk fat globules. Mol. BioSyst. 8, 967–974. 10.1039/C2MB05400K 22193558

[B65] Spertino S. Boatti L. Icardi S. Manfredi M. Cattaneo C. Marengo E. (2018). Cellulomonas fimi secretomes: *in vivo* and *in silico* approaches for the lignocellulose bioconversion. J Biotechnol. 270, 21–29. 10.1016/j.jbiotec.2018.01.018 29409863

[B66] Schluttenhofer C. Yuan L. (2017). Challenges towards revitalizing hemp: a multifaceted crop. Trends Plant Sci. 22 (11), 917–929. 10.1016/j.tplants.2017.08.004 28886910

[B67] Schneider C. A. Rasband W. S. Eliceiri K. W. (2012). NIH Image to ImageJ: 25 years of image analysis. Nat. Methods 9, 671–675. 10.1038/nmeth.2089 22930834 PMC5554542

[B68] Simopoulos A. P. (2008). The importance of the omega-6/omega-3 fatty acid ratio in cardiovascular disease and other chronic diseases. Exp. Biol. Med. 233, 674–688. 10.3181/0711-MR-311 18408140

[B69] Struik P. C. Amaducci S. Bullard M. J. Stutterheim N. C. Venturi G. Cromack H. T. H. (2000). Agronomy of fibre hemp (*Cannabis sativa* L.) in Europe. Ind. Crops Prod. 11, 107–118. 10.1016/S0926-6690(99)00048-5

[B70] Tang C. H. Ten Z. Wang X. S. Yang X. Q. (2006). Physicochemical and functional properties of hemp (Cannabis sativa L.) protein isolate. J. Agric. Food Chem. 54, 8945–8950. 10.1021/jf0619176 17090145

[B71] Tang Y. Li X. Chen P. X. Zhang B. Hernandez M. Zhang H. (2015). Characterization of fatty acid, carotenoid, tocopherol/tocotrienol compositions and antioxidant activities in seeds of three Chenopodium quinoa Willd. genotypes. Food Chem. 174, 502–508. 10.1016/j.foodchem.2014.11.040 25529712

[B72] Tang K. Struik C. Yin X. Thouminot P. Bjelková M. Amaducci S. (2016). Comparing hemp (*Cannabis sativa* L.) cultivars for dual-purpose production under contrasting environments. Ind. Crops Prod. 87, 33–44. 10.1016/j.indcrop.2016.04.026

[B73] Vonapartis E. Aubin M. P. Seguin P. Mustafa A. F. Charron J. B. (2015). Seed composition of ten industrial hemp cultivars approved for production in Canada. J. Food Comp. Anal. 39, 8–12. 10.1016/j.jfca.2014.11.004

[B74] Yan X. Tang J. dos Santos Passos C. Nurisso A. Avel lo Simões-Pires C. Ji M. (2015). Characterization of lignanamides from hemp (*Cannabis sativa* L.) seed and their antioxidant and acetylcholinesterase inhibitory activities. J. Agric. Food Chem. 63, 10611–10619. 10.1021/acs.jafc.5b05282 26585089

[B75] Zengin G. Menghini L. Di Sotto A. Mancinelli R. Sisto F. Carradori S. (2018). Chromatographic analyses, *in vitro* biological activities, and cytotoxicity of *Cannabis sativa* L. essential oil: a multidisciplinary study. Molecules 23 (12) 1–26. 10.3390/molecules23123266 PMC6320915 30544765

[B76] Zhang J. L. Zhang S. B. Zhang Y. P. Kitajima K. (2015). Effects of phylogeny and climate on seed oil fatty acid composition across 747 plant species in China. Ind. Crops Prod. 63, 1–8. 10.1016/j.indcrop.2014.10.045

